# Multifunctional nanoparticle for cancer therapy

**DOI:** 10.1002/mco2.187

**Published:** 2023-01-11

**Authors:** Yan Gao, Kaiyu Wang, Jin Zhang, Xingmei Duan, Qiu Sun, Ke Men

**Affiliations:** ^1^ State Key Laboratory of Biotherapy and Cancer Center West China Hospital of Sichuan University Chengdu Sichuan Province China; ^2^ Department of Pharmacy Personalized Drug Therapy Key Laboratory of Sichuan Province Sichuan Academy of Medical Sciences & Sichuan Provincial People's Hospital School of Medicine University of Electronic Science and Technology of China Chengdu Sichuan Province China

**Keywords:** backbone, cancer, modification, multifunctional nanoparticles

## Abstract

Cancer is a complex disease associated with a combination of abnormal physiological process and exhibiting dysfunctions in multiple systems. To provide effective treatment and diagnosis for cancer, current treatment strategies simultaneously focus on various tumor targets. Based on the rapid development of nanotechnology, nanocarriers have been shown to exhibit excellent potential for cancer therapy. Compared with nanoparticles with single functions, multifunctional nanoparticles are believed to be more aggressive and potent in the context of tumor targeting. However, the development of multifunctional nanoparticles is not simply an upgraded version of the original function, but involves a sophisticated system with a proper backbone, optimized modification sites, simple preparation method, and efficient function integration. Despite this, many well‐designed multifunctional nanoparticles with promising therapeutic potential have emerged recently. Here, to give a detailed understanding and analyzation of the currently developed multifunctional nanoparticles, their platform structures with organic or inorganic backbones were systemically generalized. We emphasized on the functionalization and modification strategies, which provide additional functions to the nanoparticle. We also discussed the application combination strategies that were involved in the development of nanoformulations with functional crosstalk. This review thus provides an overview of the construction strategies and application advances of multifunctional nanoparticles.

## Graphical abstract

Cancer is a complex disease associated with a combination of abnormal physiological processes affecting multiple systems. The single nanocarriers are not satisfy complex cancer diseases. However, multifunctional nanoparticles are upgraded versions of the original function and involve a sophisticated system with a proper backbone. Here, the construction strategies and application advances of multifunctional nanoparticles are systemically summarized.

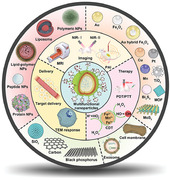

## 1 INTRODUCTION

Cancer is a complicated disease involving a combination of abnormal physiological processes affecting multiple systems, including DNA repair, apoptosis, and immune function.^1^, ^2^, ^3^ Supported by abnormal signal transduction pathways, tumor tissues simultaneously manage multiple biological activities, including proliferation, metastasis, and immune escape, rendering cancer one of the most challenging diseases to treat. Current cancer treatment strategies focus on different tumor targets, including proliferation, metastasis, and immune suppression.^4^, ^5^ Meanwhile, new technologies are also emerging to facilitate the transportation and function of therapeutic agents.

In this context, nanoparticles provide several advantages for cancer treatment and have proven efficacy in solving the delivery issue of hydrophobic agents, protecting biosensitive cargos, and promoting controlled drug‐release.^6^, ^7^, ^8^ Nanoparticles are also reliable platforms for tumor monitoring and microenvironment‐responsible technologies.^9^, ^10^ Besides, the nanoparticle carrier itself is also capable of stimulating an immune response, further showing therapeutic potential.^11^ However, cancer is complicated in terms of its mechanisms of development, which involves genes, various cells, and physiological processes. Cancer development encompasses several processes simultaneously, including proliferation, metastasis, and immune escape. In this regard, nanoparticles with single functions are not sufficient in all tumors. Therefore, there is a high demand to develop multifunctional nanoparticles.

A multifunctional nanoparticle is not simply an upgraded version of the original function. Instead, multifunctional nanoparticles integrate different functions to further expand the carrier's application, thus achieving two or more capacities.^12^, ^13^ For cancer therapy, this design can achieve tumor suppression, tracking, and microenvironment modulation, ultimately providing a more comprehensive monitoring and controlling strategy for tumor management. However, in addition to these advantages, multifunctional nanoparticles also have higher construction requirements. Except for their basic drug delivery ability, multifunctional nanoparticles must be capable of loading extra functions. The backbones of multifunctional nanoparticles are categorized as organic (polymeric, liposomes, and protein) or inorganic backbone (metal and nonmetallic), as well as biomimetic backbone (cell membranes and exosomes). Their backbones are responsible for joint external function groups or motifs with various chemical structures. Therefore, the backbones of these nanocarriers are expected to be more flexible in both the inner and surface layers, making them more amenable to covalent or noncovalent reactions. As multifunctional nanoparticles are designed to improve clinical treatment, their formulation and preparation procedures should be relatively simple. It is also challenging to have the two or more involved functions cooperate smoothly and even synergistically on one nanoplatform. With the realization of these conditions, multifunctional nanoparticles are expected to represent an efficient form of cancer therapy. Despite the above technical difficulties, many newly developed multifunctional nanoparticles have emerged in recent research. Indeed, these well‐constructed multifunctional nanocarriers have demonstrated therapeutic potential in cancer treatment with varied function design.^14^, ^15^, ^16^

**FIGURE 1:**
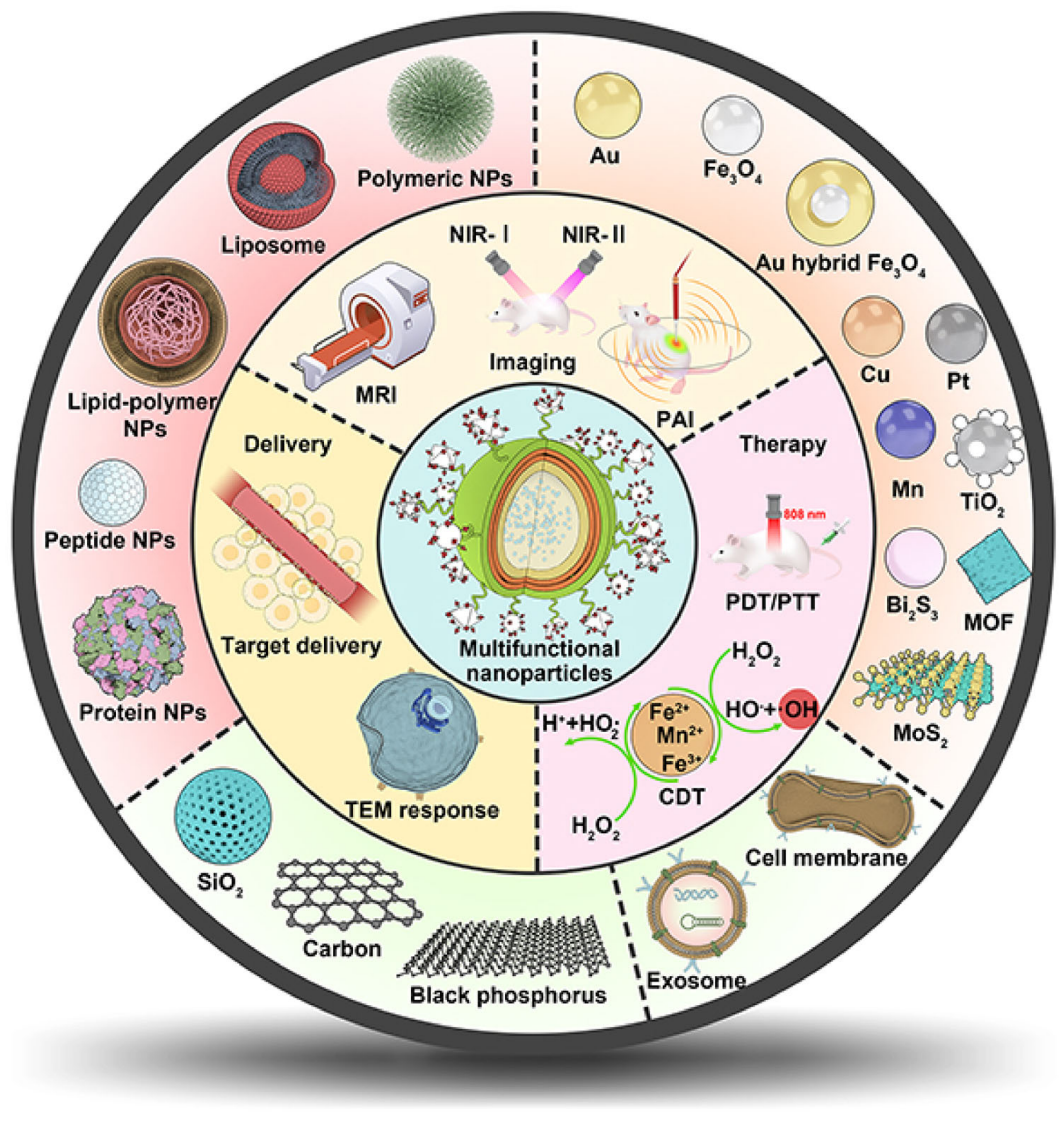
The scheme of backbone construction strategies and functional application of multifunctional nanoparticles.

In this review, we provide a comprehensive understanding and detailed analyzation of the currently developed multifunctional nanoparticles; their platform structures were summarized in the aspect of organic, inorganic, and biomimetic‐derived backbones. We especially emphasized on the functionalization and modification strategies that provide additional functions to the nanoparticle including drug delivery, in vivo imaging, and advanced therapy. We also discussed the application combination strategies that were involved in the development of discussed nanoformulations. The mechanisms that facilitate the functional crosstalk were also investigated, and their advanced applications in cancer diagnosis and treatment were highlighted (Figure 1).

## 2 BACKBONES AND STRUCTURES OF MULTIFUNCTIONAL NANOPARTICLES

According to the composition of nanoparticles, the backbones of multifunctional nanoparticles are categorized as organic (polymeric, liposomes, lipid–polymer hybrid, protein, and peptide) or inorganic backbone (Fe_3_O_4_, gold [Au], mesoporous silica, and graphene), as well as biomimetic backbone (cell membranes and exosome). Due to the plasticity and modification, these backbones are mainly prepared by covalent or noncovalent modification to obtain multifunctional nanoparticles for cancer therapy. Here, we highlight the modification strategies of organic, inorganic, and biomimetic backbones and the summary of multifunctional nanoparticle construction strategies is in Table 1.

**TABLE 1:** Summary of the multifunctional nanoparticle construction strategies

Category	Backbone	Multifunction construction strategies	Functions	References
Polymeric nanoparticles	Poly(lactic‐co‐glycolic) acid (PLGA)	Physical coating	Drug delivery/H_2_O_2_ catalytic/ Immune response/photoacoustic imaging/ Ultrasound imaging/photosensitive/ Thermal‐sensitive	^14^, ^16^, ^23^, ^24^, ^25^
		Encapsulation		
		Crosslinking with carboxyl groups		
	Polyethylene glycol (PEG)	Crosslinking with hydroxyl groups	Drug delivery/hypoxia‐sensitive/ Subsequent nuclear targeting	^33^, ^34^, ^35^
	Polydopamine (PDA)	Crosslinking with amine groups	Drug delivery/prolong blood circulation/ pH‐sensitive/photothermal‐sensitive/ Tumor targeting/MRI	^46^, ^47^, ^48^
		Crosslinking with catechol groups		
		Crosslinking with the carbon‐carbon double bonds		
	Mesoporous polydopamine	Physical absorption	Drug delivery/photothermal‐sensitive/ GSH‐sensitive/tumor targeting	^50^, ^51^
		Crosslinking with amine groups		
		Crosslinking with catechol groups		
		Crosslinking with the carbon‐carbon double bonds		
	Dendrimer	Crosslinking with terminal amine groups	Drug delivery/PET‐CT Imaging/photosensitive/ GSH‐Sensitive/H_2_O_2_‐sensitive	^62^, ^63^, ^64^, ^65^
		The inner core substitution		
		Repeating units controlling		
	Polyethyleneimine (PEI)	Crosslinking with amine groups	Drug delivery/pH‐sensitive/ ATP‐sensitive/tumor targeting/ SPECT imaging/photosensitive	^71^, ^72^, ^73^
	Cyclodextrin (CDs)	Crosslinking with hydroxyl groups	Drug delivery/redox‐sensitive/ Tumor targeting/pH‐sensitive/immune response	^15^, ^80^, ^81^, ^84^
Polymeric nanoparticles	Chitosan	Crosslinking with amino groups	Drug delivery/tumor targeting/pH‐sensitive/ Photosensitive/ultrasound‐sensitive/ Ultrasound imaging/ROS‐sensitive/ Fluorescence imaging/photothermal‐sensitive	^90^, ^91^, ^93^
		Crosslinking with hydroxyl groups		
	Dextran	Crosslinking with hydroxyl groups	Drug delivery/pH‐sensitive/ Redox‐sensitive/tumor targeting/ Immune response/photothermal‐sensitive	^101^, ^102^
	Hyaluronic acid (HA)	Crosslinking with carboxyl groups	Drug delivery/tumor targeting/ pH‐sensitive/GSH‐sensitive/ Photothermal‐sensitive	^109^, ^110^
		Crosslinking with hydroxyl groups		
Liposomes	Liposomes	Encapsulation	Drug delivery/photosensitive/ Immune response/pH‐sensitive/ Tumor targeting	^123^, ^124^, ^125^, ^126^, ^127^
		Polymerization with polymers		
		The head phospholipid substitution		
Lipid–polymer hybrid nanoparticles	Lipid–polymer hybrid	Encapsulation	Drug delivery/tumor targeting/ Photosensitive/photoacoustic imaging/ US imaging/ROS‐sensitive/ Ultrasound‐sensitive	^133^, ^134^
		Physical coating		
Protein and peptide nanoparticles	Protein	Physical adsorption	Drug delivery/fluorescence imaging/ Photothermal‐sensitive/pH‐sensitive/MRI	^148^, ^149^
		Crosslinking with carboxyl group		
	Peptide	Crosslinking with amine groups	Drug delivery/fluorescence imaging/ Tumor targeting	^142^, ^143^
		Crosslinking with carboxyl group		
Metal nanoparticles	Fe_3_O_4_	Physical adsorption	Drug delivery/magneto‐thermal sensitive/ Photothermal‐sensitive/magnetic targeting/ Fluorescence imaging/MRI/ Fenton‐like reaction	^158^, ^159^, ^161^, ^162^
		Physical coating		
		Crosslinking with carboxyl group		
	Mesoporous Fe_3_O_4_	Physical adsorption	Drug delivery/magneto‐thermal sensitive/ Photoacoustic imaging/ultrasound imaging/ MRI	^163^, ^164^
	Superparamagnetic iron oxide (SPIONs)	Drug delivery	MRI/activate ferroptosis	^167^
	Gold (Au)	Crosslinking with Au‐S bond	Drug delivery/fluorescence imaging/ Redox‐sensitive/photothermal‐sensitive/ Electrical conductivity	^169^, ^176^, ^177^, ^178^, ^179^
		Crosslinking with electrostatic interaction		
	Fe_3_O_4_/Au hybrid	Crosslinking with Au─S bonds	Drug delivery/photothermal‐sensitive/MRI/ CT imaging/pH‐sensitive/magnet‐sensitive	^184^
	Copper (Cu)	Crosslinking with coordination bond	Drug delivery/GSH‐sensitive	^190^
	CuS	Crosslinking with Cu─SH bond	Drug delivery/photothermal‐sensitive	^193^
	Hollow porous CuS	Physical absorption	Drug delivery/Tumor targeting/ Immune response	^194^
Metal nanoparticles	Titanium (TiO_2_)	Crosslinking with ionic bond	Drug delivery/immune response/ Photosensitive/Fenton‐like reaction	^204^, ^205^, ^206^
		Crosslinking with hydrogen groups		
	UiO‐66	Physical absorption	Drug delivery/pH‐sensitive/ Immunosensor/photosensitive	^216^, ^217^
		Crosslinking with carboxyl groups		
	MOF‐5	Substituting with other metal icons	Drug delivery/immune response	^218^
	MOF‐74	Crosslinking with metal‐sulfur bond	Drug delivery/conductivity/redox‐sensitive	^219^
	Platinum (Pt)	Crosslinking with coordination bonds	Drug delivery/photosensitive/ pH‐sensitive/immune response/ Tumor targeting	^233^, ^234^
		Physical absorption		
	Mesoporous Pt	Physical absorption	Drug delivery/photothermal sensitive/ CT imaging	^235^
	Molybdenum disulfide (MoS_2_)	Crosslinking with S─S bond	GSH‐sensitive/photothermal sensitive/ Tumor targeting/pH‐sensitive/PET imaging	^242^, ^246^, ^247^
		Crosslinking with C─S bond		
	Manganese atom	Encapsulation	Drug delivery/MRI/fluorescence imaging/ Fenton‐like reaction/immune response	^253^
	MnCO_3_	Physical absorption	Drug delivery/pH‐sensitive/MRI/ Ultrasound imaging	^254^
	MnO_2_	Crosslinking with polymers	Drug delivery/redox‐sensitive/ Photothermal sensitive	^255^
Metallic nanoparticles	Bismuth sulfide (Bi_2_S_3_)	Physical adsorption	Drug delivery/radiosensitive/ Tumor targeting/photoacoustic imaging/ CT imaging/photothermal‐sensitive	^269^, ^270^
		Encapsulation		
Nonmetallic nanoparticles	Mesoporous silica (MSNs)	Physical absorption	Drug delivery/tumor targeting/ Photothermal sensitive/pH‐sensitive/ Immune response	^277^, ^278^, ^279^ ^,^ ^282^, ^283^, ^284^
		Crosslinking with silanol groups		
		Physical absorption		
	Hollow mesoporous silica (HMSNs)	Encapsulation	Drug delivery/photothermal sensitive/ Fluorescence imaging/ PET imaging/ Chemiluminescence imaging	^288^
		Crosslinking with silanol groups		
	Graphene	Intercalating with other metals	Drug delivery/photosensitive/ Fluorescence imaging/MRI	^299^
		Physical absorption		
	Graphene oxide	Crosslinking with hydroxyl groups	Drug delivery/fluorescence imaging/ Photothermal‐sensitive/tumor targeting	^302^, ^303^
		Crosslinking with epoxy groups		
		Crosslinking with carboxyl groups		
		Crosslinking with carbonyl groups		
	Carbon quantum dots	Physical adsorption	Drug delivery/fluorescence imaging/MRI	^310^, ^311^
		Crosslinking with amine groups		
		Crosslinking with hydroxyl groups		
Nonmetallic nanoparticles	Black phosphorus nanosheets	Physical coating	Drug delivery/tumor targeting/ Photothermal‐sensitive/pH‐sensitive/ Immune response	^316^, ^317^, ^318^, ^319^
		Physical adsorption		
		Charge coupling		
Biomimetic nanoparticles	Cell membranes	Encapsulation	Drug delivery/photoacoustic imaging/ Photothermal imaging/ Fluorescence imaging/pH‐sensitive/ Fenton‐like reaction/ Immune response/tumor targeting	^330^, ^331^, ^332^, ^336^, ^337^
		Crosslinking with amine groups		
		Crosslinking with thiol groups		
		Crosslinking with carboxyl groups		
		Fusing with other cell membranes		
	Exosome	Encapsulation	Drug delivery/tumor targeting/ Fenton‐like reaction/immune response/ Photosensitive	^342^, ^343^
		Crosslinking with amine groups		
		Crosslinking with thiol groups		
		Crosslinking with carboxyl groups		
		Crosslinking with amine groups		

### 2.1 Organic backbone

#### 2.1.1 Polymeric nanoparticles

*PLGA*: Poly(lactic‐co‐glycolic) acid (PLGA) is a copolymer of polylactic acid and polyglycolic acid.^17^ Due to its biodegradability and biocompatibility, several drugs based on PLGA have been approved by the United States Food and Drug Administration (US FDA) for clinical cancer application, such as Lupron Depot^®^, Sandostatin Lar^®^, and Trelstar^®^.^18^, ^19^ As PLGA nanoparticles possess both hydrophilicity and hydrophobicity, they have the potential to form a water/oil/water (W/O/W) core–shell structure by the compatibility principle.^20^, ^21^ Additionally, PLGA nanoparticles possess free carboxyl end‐groups and can be modified as multifunctional nanoparticles, by connecting other bioactivation agents through covalent bonds.^22^ Based on the delivered ability of PLGA, there are three main modification strategies for cancer therapy, namely encapsulation, coating, and covalent modification. Initially, these water‐soluble agents can be encapsulated in the PLGA core, while the hydrophobic agents are loaded in the PLGA shell, forming a core–shell structure. Chen et al.^14^ used water‐soluble catalase (Cat) and hydrophobic imiquimod (R837) to prepare multifunctional nanoparticles based on PLGA (PLGA–R837@Cat). The Cat was encapsulated in the PLGA core and R837 was loaded in the PLGA shell. Under X‐ray radiation, these nanoparticles can decompose H_2_O_2_ to generate O_2_ to relieve tumor hypoxia and activate the immune response. In this work, they used the classic shell–core structure of PLGA, the Cat and drugs were coencapsulated and formed multifunctional PLGA nanoparticles to realize the immunotherapy of cancer. Similarly, Li et al.^23^ reported that PLGA nanoparticles encapsulated with sinoporphyrin sodium and perfluoropentane (PFP) in the core, and IR780 loaded in the shell form multifunctional nanoparticles (DIPP‐nanoparticles) for achieving cancer treatment with dual‐mode imaging. Additionally, these bioactivation agents can be coated on the surface of PLGA nanoparticles to provide the function of coating substances, such as polydopamine (PDA). Indeed, Peng et al.^24^ prepared a multifunctional near‐infrared responsive material based on PLGA nanoparticles (docetaxel [DTX]–PLGA nanoparticles@PDA‐TPGS), in which PLGA was loaded with DTX and coated with PDA by an oxidative polymerization reaction. After intravenous treatment with these nanoparticles, the tumor was suppressed under chemo‐photothermal therapy (PTT). Furthermore, multifunctional PLGA nanoparticles can also be prepared by direct covalent, forming of a block copolymer, such as PLGA–PEG. Yu et al.^25^ reported that PEG and peptide functional‐modified PLGA nanoparticles could be used to deliver IR780, forming aNP@IR780. PLGA–PEG–maleimide was loaded with IR780 and then conjugated with PD‐L1 binding peptide; as a result, aNP@IR780 showed a strong CT26 antitumor effect by activating immune cells following near‐infrared irradiation (NIR). Additionally, Zhou et al.^16^ reported that using PLGA–PEG–poly(N‐isopropylacrylamide) and PEG–PLGA–biotin to deliver photochemical agent (ATT‐2), forming PATA nanoparticles, facilitated effective tumor targeting and the property of phase transition temperature. Moreover, PLGA nanoparticles can be also modified by target peptide to enhance cancer therapy or by codelivery with magnetic nanoparticles for increasing T_2_ contrast magnetic resonance imaging (MRI).^26^

*PEG*: Polyethylene glycol (PEG), also known as polyethylene oxide and polyoxyethylene, has a linear polymer structure.^27^, ^28^ PEG nanoparticles can prolong the systemic circulation time of the drug in the body and reduce immunogenicity, resulting in their wide use in cancer therapy.^29^ Due to their nonimmunogenic and biodegradable properties, PEG nanoparticles are regarded as a “stealth” polymer and several, including DOXil^®^, Asclera^®^, and Movantik^®^, are approved by the US FDA.^30^, ^31^, ^32^ Multifunctional modification strategies of PEG nanoparticles are mainly conducted to activate the two terminal hydroxyl groups and change the structure to introduce functional groups. To avoid the formation of self‐crosslinking complexes, PEG with only one terminal hydroxyl group is synthesized, and another terminal hydroxyl group is modified by other functional groups, including the methoxy (MeO) group and amine groups. For example, Liu et al.^33^ prepared tumor‐targeted IDM nanomedicine based on a PEG block copolymer. To this end, they used MeO–PEG–NH_2_ conjugated with β‐benzyl‐_L_‐aspartate N‐carboxy‐anhydride form PEG‐*b*‐PBLA and modified it with 6‐(2‐nitroimidazole) hexylamine to codeliver with doxorubicin (DOX) and indocyanine green (ICG) to efficiently suppress 4T1 cancer by inducing an immunogenic response and PDT/PTT. Multifunctional modification of hydroxyl groups at the both ends of PEG realized tumor immunotherapy and photodynamic therapy (PDT). The terminal hydroxyl group of MeO–PEG (mPEG) is considered to be active at the COOH for covalent binding to hydroxy‐containing drugs, forming a pH‐sensitive ester bond. Jing et al.^34^ prepared multifunctional nanoparticles (PECL/DA‐Tat‐M) based on the PEG backbone. The amine groups on NH_2_–mPEG–PCL were conjugated with the carboxyl terminal groups on Tat peptide, and modified by 2,3‐dimethylmaleic anhydride (DA) for targeting 4T1 breast tumors with a pH response. Additionally, the structure of PEG can be changed to form PEG derivatives, such as 8‐arm PEGs. Indeed, Zhang et al. prepared a new PEG–Ce6–Fe^2+^‐gossypol metal‐phenolic networks (PFGs) based on the PEG backbone.^35^ To this end, they used 8‐arm PEG–succinimidy‐(N‐methyl‐polyethylene glycol (NHS groups) to conjugate with Ce6, Fe^2+^, and gossypol, forming PFGs to efficiently inhibit tumor growth by combining with chemo‐photodynamic therapy (PDT). In other studies, PEG was modified by bifunctional groups for covalent binding to other bioactive agents to enhance target cancer therapy, such as NHS–PEG–mal, NH_2_–PEG–mal, and mal–PEG–COOH.^36^, ^37^, ^38^

*PDA*: PDA nanoparticles are prepared by the oxidative polymerization of dopamine hydrochloride.^39^ Given the abundance of catechol, quinine, and amine groups in the structure, PDA nanoparticles exhibit hydrophilic properties and are easy to modify.^40^, ^41^ Due to the advantages of excellent biocompatibility and photothermal conversion ability, PDA nanoparticles are regarded as a near‐infrared absorbing materials and are widely applied in cancer therapy.^42^, ^43^, ^44^ Based on the dopamine hydrochloride structure of PDA, the modification design strategies of PDA involve active catechol groups, amine groups, and carbon–carbon double bonds by covalent modification.^45^ Specifically, the carbon–carbon double bonds in PDA nanoparticles have the potential to be covalently modified by reactants containing amine groups by Schiff base reaction, such as PEG–NH_2_. Indeed, Zhu et al.^46^ developed a multifunctional nanoplatform based on PDA nanoparticles (Fe(III)PP@SAS nanoparticles), in which the PDA nanoparticles were covalently modified by mPEG–NH_2_ by a Schiff base reaction to load with FeCl_3_·6H_2_O and sulfasalazine. These nanoparticles can trigger the cancer cell ferroptosis, with pH‐sensitive and near‐infrared light irradiation. Fe(III)PP@SAS nanoparticles utilized the carbon–carbon double bonds in PDA with covalent modification and realized multifunctional PDA nanoparticles treated cancer with photo‐ferrotherapy. Moreover, the high number of double bonds in PDA can be covalently modified by reactants containing thiol groups (SH) using the Michael addition reaction for further modification, such as mPEG–SH. Zhou et al.^47^ reported a dual peptide (RGD and beclin 1) and mPEG–SH functional modified by PDA to obtain PPBR nanoparticles for targeting tumor cells by recognition of α_v_β_3_ receptors and causing cell autophagy by beclin 1 peptide. Additionally, due to the catechol structure, PDA can directly conjugate with metal or nonmetal ions by coordinate covalent bonds, such as Fe. Wang et al.^48^ prepared multifunctional PDA nanoparticles doped with ferric ion and conjugated with alendronate (ALN) to form PDA/Fe–ALN nanoparticles. The catechol groups on PDA are centered on ferric ions by coordinate covalent bonds and are conjugated with the amino groups on ALN. The doping with Fe to generate PDA/Fe–ALN nanoparticles achieved successful MRI for bone tumors.

Mesoporous PDA is a mesoporous structure that was first introduced based on PDA nanoparticles. Compared with PDA nanoparticles, mesoporous PDA can load multiple drugs into the porous structure. Mesoporous PDA has significantly improved drug loading capacity and high photothermal conversion efficiency, which provides a new idea for developing multifunctional PDA nanoparticles.^49^ Based on the mesoporous structure of PDA, the multifunctional design strategies of mesoporous PDA mainly include codelivery of multiple drugs and covalent modifications with catechol, amine groups, and carbon–carbon double bonds. Typically, mesoporous PDA can codeliver two or more drugs by absorption in a porous channel modified by SH groups. Hu et al.^50^ reported that using folic acid (FA) PEG thiol (FA–PEG–SH) modified on the surface of mesoporous PDA (MPPD), and codelivered with perfluorooctane and IR820 in the porous channels could enhance the cellular uptake with GSH response. Similarly, Dai et al.^51^ reported that mesoporous PDA could also be modified by AS1411 aptamer and deliver DTX for targeting prostate cancer cells with chemo‐PTT. Furthermore, other substances can be used to coat the surface of PDA based on its strong adhesion property; this would serve to improve cancer therapy with PDT, fluorescence optical imaging, and MRI guidance, such as cationic coating layer, covalent organic frameworks, and metal ions.^44^, ^52^, ^53^

*Dendrimers*: Dendrimers are nanosized polymeric nanoparticles composed of an inner core, repeating units, and terminal functional groups, forming three‐dimensional (3D) spherical nanoparticles with a regular dendritic structure.^54^, ^55^, ^56^ Dendrimers can load various hydrophobic drugs through electrostatic interactions and hydrophilic drugs with covalent bonds or chelation, which can enhance the solubility of drugs and is widely used in cancer therapy.^57^, ^58^ Based on the delivery capability of the dendrimer, multifunctional dendrimer nanoplatforms can be designed by modifying terminal functional groups, changing the inner core, and altering the controllability of the number of repeating units.^59^, ^60^ Poly(amidoamine) (PAMAM) is a commonly used dendrimer, and the terminal amine groups of PAMAM can be conjugated with NHS groups and carboxyl.^61^ Indeed, Konopka et al.^62^ prepared a multimodal tracer (^64^Cu–Rho–G4–CML) based on the PAMAM G4 dendrimer. The amine groups of the PAMAM G4 dendrimer modified by NOTA chelator, amine‐reactive tetramethyl rhodamine, and Nε‐(carboxymethyl) lysine via NHS groups. The prepared ^64^Cu–Rho–G4–CML nanotracer could target the RAGE ligand and rapidly clear in blood for optical and positron emission tomography (PET) imaging. They prepared multifunctional dendrimers by covalently modifying the amine group at the end of PAMAM to realize PET imaging‐guided tumor therapy. Similarly, Nabi et al.^63^ reported the preparation of ^67^Ga‐DTPAPAM–PEG/GEF@MUC‐1 to achieve imaging guiding of breast cancer. In this case, the amine groups of PAMAM G2 were conjugated with the carboxyl groups in diethylenetriaminepentaacetic acid through a condensation reaction, before being modified by PEG2000‐Br to load gefitinib (GEF) and chelate with ^67^Ga to selectively kill cancer cells with a combination of chemotherapy to provide an imaging guide for breast cancer. Additionally, multifunctional dendrimers can be synthesized with a peptide core, such as glutamic acid and lysine. Indeed, Zhou et al.^64^ synthesized multifunctional dendrimer nanoparticles ((PBA4–E2E)_2_–PpIX–LA2) based on a peptide. This study synthesized a G2 glutamic acid dendrimer and reacted it with protoporphyrin IX to form the (OtBu4–E2E)_2_–PpIX compound, which was modified by primary amine‐lipoic acid and conjugated with pinacol boronic ester to load paclitaxel (PTX). These dendrimers could control the drug release by GSH and H_2_O_2_ in response to cancer therapy. Additionally, Zheng et al.^65^ designed a PEGylated dendritic peptide conjugate (PDPP) based on glutamic acid and lysine to kill cancer cells under PDT. However, compared with multifunctional nanoparticles of PLGA and PEG, dendrimers are poor drug loading, have cellular toxicity, and high cost in covalently modification synthesis. Therefore, dendrimers should overcome the above disadvantages and construct multifunctional modification dendrimers nanoparticles with biocompatibility, safety, and easy synthesis properties for cancer therapy.

*PEI*: Polyethyleneimine (PEI) is a cationic polymeric that consists of repeating ethylamine units with linear and branched types.^66^, ^67^ Due to the strong positive charge on the surface of PEI, it is possible to condense nucleic acid by electrostatic interactions, facilitating high drug delivery efficiency for cancer therapy.^68^, ^69^ The multifunctional construction methods based on PEI are mainly mediated by covalent coupling amine groups.^70^ For example, the amine groups of PEI can be covalently coupled with carboxyl, such as 4‐(bromomethyl) phenylboronic acid (PBA). Ma et al.^71^ reported that using PBA, Cetuximab (C225), and DA multifunctionally modified PEI, forming PEI–DMA–C225. The amine groups in PEI were modified by PBA via a substitution reaction to form PEI–PBA. After being conjugated with C225, these nanoparticles exhibited both a pH response and ATP response. After treatment with miR146a by tail vein injection, these nanoparticles could target androgen‐independent prostate cancer and inhibit the growth of tumor cells. PEI–DMA–C225 nanoparticles utilized the amine groups of PEI to multifunctional modified with other bioactive groups, which added the capability of tumor environment response in PEI‐based nanoparticles. Additionally, the amine groups of PEI could be covalently coupled with NHS groups, such as mPEG–NHS. Indeed, Zhu et al.^72^ prepared PEI with alkoxyphenyl acylsulfonamide (APAS) and ^131^I to form multifunctional nanoparticles (APAS–^131^I‐PNPs/DOX). The amine groups in PEI were conjugated with mPEG–NHS and APAS to improve the cancer cell uptake. By loading DOX and labeling ^131^I, the prepared nanoparticles inhibited cancer cells in combination with chemotherapy and radiotherapy. Moreover, PEI can be self‐assembled with other polymeric nanoparticles to form block polymers, such as PEI–PCL. Wang et al.^73^ prepared MPPD@IR825 nanoparticles based on poly (ethylene imine)‐poly(ε‐caprolactone) block polymers (PEI–PCL). The dimethyl maleic anhydride‐modified PEGylated shell could increase the drug release of DOX and IR825; these nanoparticles exhibited efficient breast tumor ablation with chemo‐PTT.

*CDs*: Cyclodextrins (CDs) are degradation products of amylase and consist of glucopyranose forming cyclic oligosaccharides.^74^ CDs are widely applied as delivery vectors for cancer therapy because of their hydrophilic properties and ability to form host‐guest structures with drugs.^75^, ^76^, ^77^ Due to the multiple glucopyranoside units in the structure of CDs, the multifunctional design strategies based on CDs mainly modify the free hydroxyl groups by covalent coupling.^78^ β‐Cyclodextrin (β‐CD) is a commonly used CD with seven glucopyranose units, while the free hydroxyl groups can be activated in carboxyl groups such as N, N′‐carbonyl diimidazole (CDI).^79^ Mousazadeh et al.^80^ prepared novel redox‐sensitive folate‐appended‐polyethylenimine‐β‐CD host‐guest supramolecular nanoparticles (HGSNP) for pH‐dependent sustained intracellular drug release and simultaneous efficient gene transfection. The hydroxyl groups in β‐CD were activated by CDI, forming carboxyl groups and conjugated with the amine groups in PEI to modify FA and load with DOX, forming HGSNP. The prepared HGSNP nanoparticles used the free hydroxyl groups in β‐CD to multifunctional modified with FA, which realized the tumor targeting and pH response for cancer therapy. Additionally, the hydroxyl groups of β‐CD could be covalently coupled with carbonyl groups through nucleophilic substitution. Similarly, Zhang et al.^15^ also used CDI to active the hydroxyl groups in β‐CD to conjugate to the amino groups of PBA pinacol ester and self‐assembled with DSPE–PEG to obtain OCD NP to regulate the proinflammatory microenvironment and target colon cancer. Moreover, the hydroxyl groups of β‐CD could be activated by anhydride in order to introduce carboxyl groups, such as succinic anhydride. Rodell et al.^81^ used succinic anhydride to activate β‐CD in carboxyl groups to conjugate with l‐lysine for delivery of the immune agonist R848 to achieve cancer immunotherapy. However, carboxymethyl–β‐CD (CM–β‐CD) is a CD derivative formed by connecting the carboxymethyl group to the CD cavity through an ether bond.^82^ The carboxymethyl group is linked to the edge of the CD cavity through the ether bond, which can be dissociated to carboxyl groups for covalent coupling, such as to amine groups in cell‐penetrating peptide.^83^ Wei et al.^84^ reported using a pH‐responsive cell‐penetrating peptide (R6H4)‐modified CM–β‐CD obtained by a condensation reaction and loaded curcumin (CUR) to form multifunctional RCC nanoparticles. These nanoparticles could target delivery and enhance the CUR anticancer effects safely and with no toxicity.

*CS*: Chitosan (CS) is a natural cationic polymer consisting of β‐(1,4)‐linked N‐acetyl glucosamine units prepared by deacetylation of chitin in crustacean shells.^85^, ^86^ As CS nanoparticles have excellent biocompatibility and biodegradability, they can be used as a drug coating and drug delivery vector for cancer therapy.^87^, ^88^ Based on the N‐acetyl glucosamine unit structure in CS, multifunctional design strategies of CS are active free hydroxyl and amino groups by conjugating with other agents.^89^ The active amino groups in CS can be covalently coupled with carboxyl groups through an amidation reaction. For example, Zhang et al.^90^ used glycol‐modified CS (GCP) to conjugate with pyropheophorbide a (Ppa) and DOTA via an amidation reaction to form GCP‐nanoparticles. They used radionuclide ^99^mTc/^177^Lu chelation with DOTA to realize radiotherapy. These nanoparticles could inhibit the growth of cancer cells in 4T1 tumor‐bearing mice by laser irradiation. They utilized the free hydroxyl and amino groups in CS multifunctional modified with photosensitizes and radionuclide, which realized photodynamic radionuclide therapy of cancer based on CS nanoparticles. Additionally, the active amino groups in CS could be covalently coupled with NHS groups. Zhao et al.^91^ prepared an ultrasound‐responsive multifunctional nanoparticle based on CS nanoparticles (SP94–DOX–NDs) for castration‐resistant prostate cancer. With the help of a Mal–PEG2000–NHS linker, CS was conjugated with a SP94‐HS peptide via a sulfhydryl‐maleimide coupling reaction, forming SP94–PEG2000–CS to deliver DOX for effective anticancer treatment, with potential for real‐time ultrasound imaging. Moreover, carboxymethyl CS, the carboxymethylated product of CS, adds a carboxyl group to CS, providing another modification method for multifunction, such as nucleophilic substitution.^92^ Shao et al.^93^ devised a multifunctional nanosystem (CMCh–BAPE–RGD@ICG) for targeted and image‐guided PTT in gastric cancer. They used carboxymethyl CS (CMCh) to couple with 4‐hydroxymethyl‐pinacol phenyl borate (BAPE) to form an amide bond by bromination and nucleophilic substitution to obtain CMCh–BAPE nanoparticles for effectively inhibiting the tumor growth of gastric cancer under ICG‐mediated near infrared imaging and PTT.

*Dextran*: Dextran is a hydrophilic natural polymer formed by the condensation of glucose.^94^ The polymer backbone consists of α‐1,6 glycosidic bonds between glucose monomers.^95^ Similar to other natural polymers, with hydrophilic, biocompatible, and biodegradable characteristics, dextran can be widely used as scaffold, surface coating, and delivery vector for cancer therapy.^96^, ^97^, ^98^, ^99^ Due to the glucose monomers in dextran, multifunctional strategies based on dextran mainly involve chemical modification of the active hydroxyl groups, which can increase the drug release by stimuli‐responsive activity, combining immunotherapy and PTT for cancer.^100^ For example, the active hydroxyl groups in dextran can be covalently coupled with carboxyl groups. Indeed, Curcio et al. prepared nanoparticles (DFNPs) based on two new amphiphilic dextran derivatives.^101^ Condensation of cysteamine‐modified dextran (DEX) with PEG_600_COOH could be conducted to obtain DEXssPEGCOOH. As DEXssPEGCOOH consists of hydrophobic chemicals with disulfide bridges, hydrazone, or imine, these nanoparticles showed stimuli‐responsive activity to achieve the pH and redox‐responsive release of anticancer drugs. The prepared DFNPs used active hydroxyl groups in dextran to multifunctional modified with bioactive agents by covalently coupling, which realized the targeting and stimuli‐responsive activity for cancer therapy. Additionally, the active hydroxyl groups in dextran can be covalently coupled with aldehyde groups via an acetal reaction. Gao et al.^102^ reported the synthesis of a multifunctional vaccine (I‐R‐Ap‐AcDEX nanoparticles) based on dextran for treating cancer with immunotherapy and PTT. The hydroxyl groups of dextran and 2‐methoxypropene via an acetal reaction formed cyclic and acyclic acetals to obtain AcDEX nanoparticles, which were then conjugated with ICG, imiquimod (R837), and antigen peptide (Ap) to treat cancer.

*HA*: Hyaluronic acid (HA) is a natural polymer composed of d‐glucuronic acid and N‐acetyl‐d‐glucosamine.^103^ HA is a biodegradable hydrophilic polymer material that is used as a biological scaffold for cancer therapy.^104^, ^105^ Because of its tumor targeting, nonimmunogenicity, and biosafety properties, HA has been widely used in drug delivery systems for drug control and sustained release.^106^, ^107^ Due to the glucuronic acid and glucosamine in HA, multifunctional design strategies based on HA are chemically modified by active hydroxyl and carboxyl groups.^108^ For instance, the active hydroxyl and carboxyl groups can be separately conjugated with carboxyl and hydroxyl groups. Liu et al.^109^ synthesized multitarget and pH‐sensitive nanoactiniaes to codeliver icariin (ICA) and curcumin (Cur) for breast cancer. The carboxyl groups in oligomeric HA were conjugated with the amine groups in adipic dihydrazide. The hydroxyl groups in oligomeric HA were modified by biotin to load with ICA and Cur. The prepared nanoparticles had a pH‐sensitive hydrazone bond group that enabled ICA and Cur to accumulate in the tumor tissue and cancer stem cells. In this work, HA was multifunctional modified with carboxyl groups to form pH‐sensitive groups and realize tumor environment response for cancer therapy. Similarly, Yang et al.^110^ designed GSH‐responsive nanomicelles (IR780/PTX/FHSV) to encapsulate PTX and the photosensitizer IR780 iodide. The synthetic amphiphilic HA derivative (FHSV) were self‐assembled into nanomicelles in an aqueous medium. Then, PTX and IR780 were loaded into the nanomicelles to form IR780/PTX/FHSV micelles. The prepared micelles could effectively enter tumor cells and kill cancer cells by producing reactive oxygen species (ROS) and IR780‐guided PTT.

#### 2.1.2 Liposomes

**FIGURE 2:**
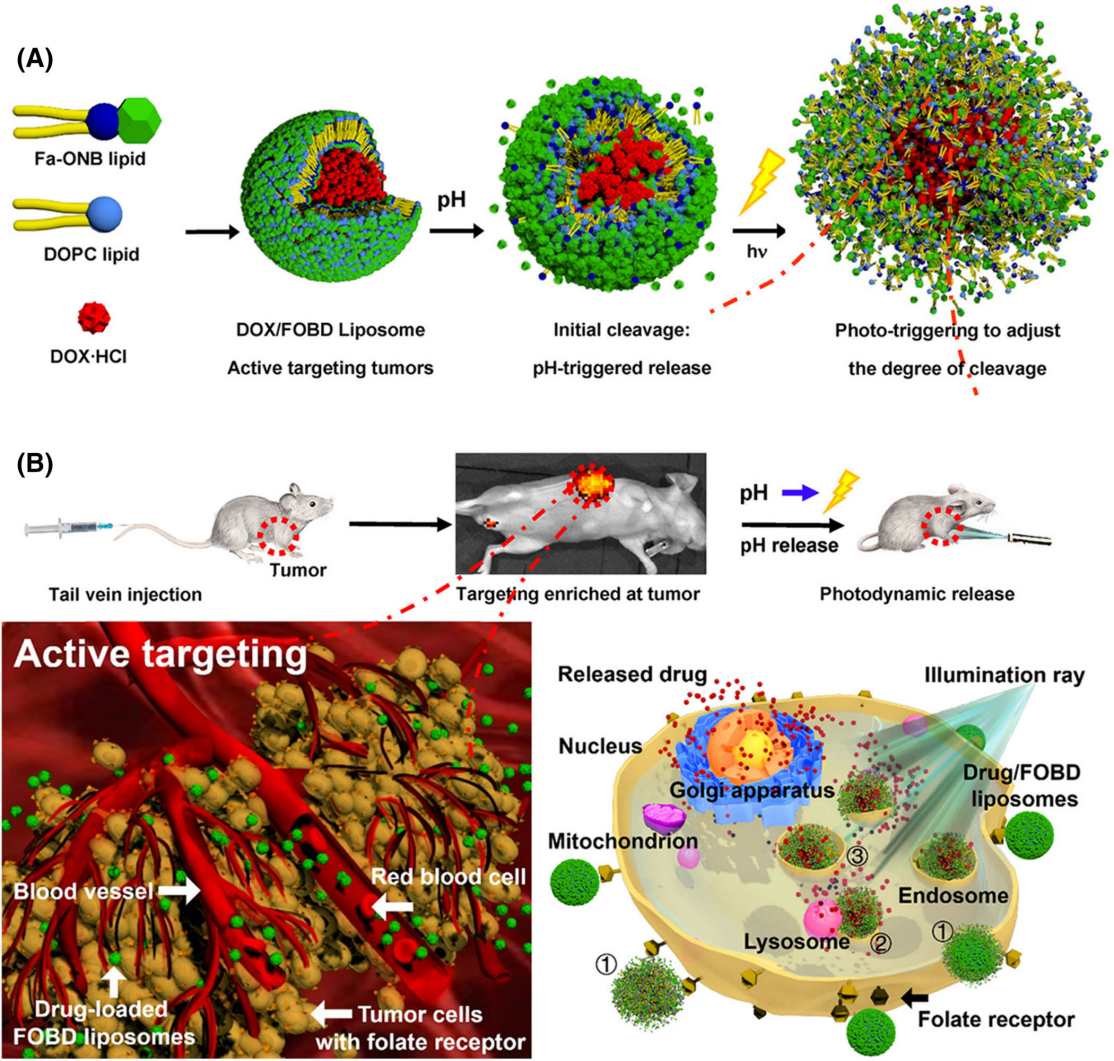
Scheme illustrating of a multifunctional nanoplatform (FOBD) using folic acid‐modified *o*‐nitro‐benzyl ester lipid for delivery of DOX‐targeted therapy in cancer. (A) Fa–ONB lipid were compounded with DOPC lipid by self‐assembly and loaded with DOX to form DOX/FOBD liposomes. These liposomes could enable dual (pH and light) triggering reactions of drug release. (B) After injected by tail vein, DOX/FOBD liposomes could target enriched at tumor site and quickly release drugs by pH response and irradiating UV light. Reprinted with permission from Ref. [127], Copyright 2020 American Chemical Society.

Liposomes are spherical vesicles with a bilayer structure composed of phospholipids.^111^ The structure of phospholipids contains a hydrophilic head consisting of a phosphate group and a quaternary ammonium salt group, as well as a lipophilic tail consisting of two longer hydrocarbon groups.^112^, ^113^ Liposomes have excellent compatibility with cells and are widely applied in drug delivery systems, and PEGylated liposomes are approved by the US FDA.^114^, ^115^ The components of multifunctional liposomes commonly include cholesterol, 1,2‐distearoyl‐*sn‐glycero*‐3‐phosphoethanolamine (DSPE), 1,2‐dipalmitoyl‐*sn‐glycero*‐3‐phosphocholine (DPPC), 1,2‐distearoyl‐*sn‐glycero*‐3‐phosphocholine (DSPC), dioleoylphosphatydic acid (DOPA), 1,2‐dioleoyl‐*sn‐glycero*‐3‐phosphocholine (DOPC), and so on. These phospholipids are used in combination to increase stability of liposomes for multifunctional nanoparticle platforms, such as cholesterol/DSPC, cholesterol/DOPA, and DSPE/DOPC/DPPC.^116^, ^117^, ^118^ Due to the amphipathic structure of phospholipids, the multifunctional modification strategies based on liposomes mainly include physical encapsulation, chemical conjugation, and changes in the type of phospholipid head.^119^, ^120^, ^121^, ^122^ Liposomes can encapsulate drugs in the inner water cavity or insert the drugs into a bilayer, forming a nanoparticle by self‐assembly, such as a photosensitizer. For example, Liu et al.^123^ designed a biomimetic liposomal system (nano‐Pt/VP@MLipo) for cancer treatment in which they loaded Pt nanoparticles in the liposome core, and encapsulated the photosensitizer Vitipophan (VP) in the liposome bilayer. These nanoparticles were coated with the RAW264.7 cell membrane, which could enhance the anticancer effect through VP‐mediated PDT. Taking the advantage of the biocompatibility of liposomes, the multifunctional modification of cell membranes endows liposomes with biomimetic properties and tumor targeting, while encapsulates photosensitizers to achieve PDT of cancer. Additionally, Ding et al.^124^ prepared a multifunctional nanoparticle (LIC) to inhibit the PI3Kγ–AKT signaling pathway and function as a PDT under light irradiation by inserting an inhibitor and photosensitizer in the liposome bilayer. Additionally, liposomes can be grafted with other polymeric nanoparticles forming block copolymers to prolong the circulation time and coupling with other bioactive agents, such as DSPE–PEG–COOH. Gu et al.^125^ developed a multifunctional nanoparticle (CP@NP‐cRGD) based on the DSPE backbone for codelivery with calcium chloroquine (CQ) and PD173074. DSPE–PEG2K–COOH was modified by cRGD by an amidation reaction to codeliver CQ and PD173074 to target pH‐sensitive cancer. Similarly, Zhao et al.^126^ used PLGA_12K_–hyd–mPEG_2K_ and DSPE–PEG_1K_–MAL to encapsulate metformin (MET), chlorin e6 (Ce6), and perfluorhexane (PFH) to effectively target cancer cells and suppress tumor growth by combining PDT and immunotherapy. Moreover, liposomes can also be synthesized with hydrophilic head and functional groups with environmentally responsive ability, such as o‐nitro‐benzyl ester. Indeed, Liu et al.^127^ developed a functional lipid (Fa–ONB) with FA and o‐nitro‐benzyl ester lipids (Figure 2). They conjugated activated FA to a lipid scaffold prepared from 4‐(bromomethyl)‐3‐nitro‐benzoic acid and didodecylamine by an amidation reaction to obtain the Fa–ONB lipid. The o‐nitro‐benzyl ester lipid enabled dual (pH and light) triggering reactions of drug release, while the FA could actively target the tumor tissue. However, the solubility and stability of liposomes are poor, which is not conducive to multifunctional modification. In addition, drugs encapsulated in liposomes are prone to leakage, and the preparation cost of liposomes are relatively high.

#### 2.1.3 Lipid–polymer hybrid nanoparticles

Lipid–polymer hybrid nanoparticles mainly consist of a polymer core and lipid shell, forming a nanoparticle complex.^128^, ^129^ The polymeric nanoparticles can encapsulate active agents forming the core, with the lipid coated as a shell to increase the drug loading capacity.^130^ Lipid–polymer hybrid nanoparticles have been widely used in drug delivery for cancer therapy because of their stability and biocompatibility.^130^, ^131^ Due to the structural diversity of polymeric nanoparticles and liposomes, multifunctional modification strategies based on lipid–polymer hybrid nanoparticles can be used to deliver bioactive agents by physical capsulation forming a core–shell structure.^132^ For example, the hydrophobicity of a photosensitizer or sonosensitizer is encapsulated in a polymer core and coated with a target peptide‐modified lipid shell. Chen et al.^133^ prepared folate‐targeted lipid–polymer hybrid nanoparticles (LPH nanoparticles) loaded with ICG and perfluoropentane (PFP) for photo‐sonodynamic therapy (SDT). The lipid film was prepared by DPPC, DPPG, DSPE–PEG‐(2000)–FA, and cholesterol to form a lipid shell, while the PLGA polymer core was added through solution evaporation. LPH nanoparticles can target tumor cells with PA imaging guidance. LPH nanoparticles utilize the complementary characteristics of polymer and liposome to codeliver photosensitizers and sonosensitizers, while being modified with targeting receptors. These modifications achieve multifunctional lipid–polymer hybrid nanoparticles tumor targeting and dual‐mode imaging guidance for cancer. Additionally, NIR dye and drugs can be encapsulated in a polymer core coated with multiple mixed lipid thin film. Zhang et al.^134^ reported the use of a B‐PDEAEA/DNA@IR780‐liposome to achieve efficient gene delivery and antitumor effects in pancreatic cancer via the binding of ROS‐response polymer core and IR780‐loaded liposome coating. Additionally, the polymer core can be designed with environment‐response materials, while the lipid shell may also be modified by PEG to increase the stability and prolong the circulation time of drug release and loading with a sonosensitizer for cancer phototherapy.^135^, ^136^

#### 2.1.4 Peptides and protein nanoparticles

Peptide nanoparticles consist of natural or synthetic amino acids the size of nanoparticles.^137^ Peptide nanoparticles are widely applied in cancer therapy, gene delivery, and target drug delivery because of the biodegradation capability of peptides.^138^, ^139^ Due to the active groups on the side chain of peptides, multifunctional design strategies based on peptide nanoparticles include physical adsorption and chemical covalent coupling.^140^, ^141^ For example, peptides contain benzene or macrocyclic structures that allow peptide nanoparticles to interact with various metals through π–π stacking or electrostatic interactions. Fan et al.^142^ assembled fluorescent peptide nanoparticles (RGD–f‐PNPs/EPI) by cyclic peptides and modified them with RGD to encapsulate epirubicin (EPI) forming a multifunctional RGD–f‐PNPs/EPI complex through π–π stacking and electrostatic interactions. Due to the surface modification of RGD, the prepared nanoparticles selectively targeted EC cells and promoted EPI internalization. These nanoparticles could be monitored by near‐infrared fluorescence and were shown to have therapeutic effects against esophageal cancer.

RGD–f‐PNPs/EPI nanoparticles utilized fluorescent peptide with cyclo[‐(_D_‐Ala‐L‐Glu‐D‐Ala‐L‐Trp)_2_‐] sequence to creatively realize the potential of visible light and near‐infrared molecular imaging, which provided a new idea for the construction of multifunctional peptide nanoparticles. Additionally, peptide side chains comprise numerous free carboxyl groups that can be covalently conjugated with amino groups. Guo et al.^143^ synthesized multifunctional dipeptide nanoparticles (DNPs nanoparticles) chelating Trp–Phe dipeptide with zinc ions. The carboxyl groups of DNPs nanoparticles were conjugated with clofarabine, aptamers AS1411, and influenza hemagglutinin peptide (HA) to codeliver siRNA and DOX to improve endosomal escape and biological imaging.

Proteins also have ideal multifunctional backbones with modification strategies that have been widely used in drug delivery for various cancer treatments because of their biocompatibility and biodegradability.^144^ As various amino acids consist of protein, multifunctional modification strategies based on protein nanoparticles include physical capsulation and chemical covalent conjugation.^145^ Albumin is a commonly used protein nanoparticle for cancer therapy due to its high drug loading efficiency, which bovine serum albumin (BSA) and human serum albumin (HSA) are commonly used in drug delivery system.^146^ There are multiple domains in the structure of albumin and different domains can bind with the different properties of drugs. Both BSA and HSA have three identical domains, and HSA has two major albumin binding sites, known as Sudlow site I and II, which can efficiently bind with hydrophobic drugs and deliver to the cancer site.^147^ Due to the functional groups of the side chain, multifunctional albumin nanoparticles could include modified amine groups with carboxyl groups.^139^ For example, Ding et al.^148^ synthesized a multifunctional nanoplatform (BITT@BSA–DSP nanoparticles) based on albumin to encapsulate the cisplatin (IV) prodrug. They oxidized cisplatin (IV) with H_2_O_2_ and reacted it with succinic anhydride to produce Pt(IV)–COOH (DSP). The carboxyl groups of DSP covalently reacted with BSA and were guided by AIEgen (BITT) to self‐assemble, forming BITT@BSA–DSP nanoparticles that enhanced the chemotherapy effect of cisplatin and inhibited the growth of bladder cancer cells. Additionally, the amine groups in albumin nanoparticles can be functional modified by NHS ester. Zhang et al.^149^ designed a multifunctional nanosystem (MnAs/ICG/HSA–RGD) based on HSA. After the coordination reaction between cRGD‐labeled HSA and manganese (Mn) chloride, ICG and arsenite were encapsulated to form MnAs/ICG/HSA–RGD; these nanoparticles released arsenite in a pH‐responsive manner and significant inhibited the growth of liver tumor cells under the combination of ICG‐guided‐PTT and chemotherapy. However, protein nanoparticles are still worthy of improvement in maintaining protein activity, reducing immunogenicity, and improving stability.

### 2.2 Inorganic backbone

#### 2.2.1 Metal nanoparticles

**FIGURE 3:**
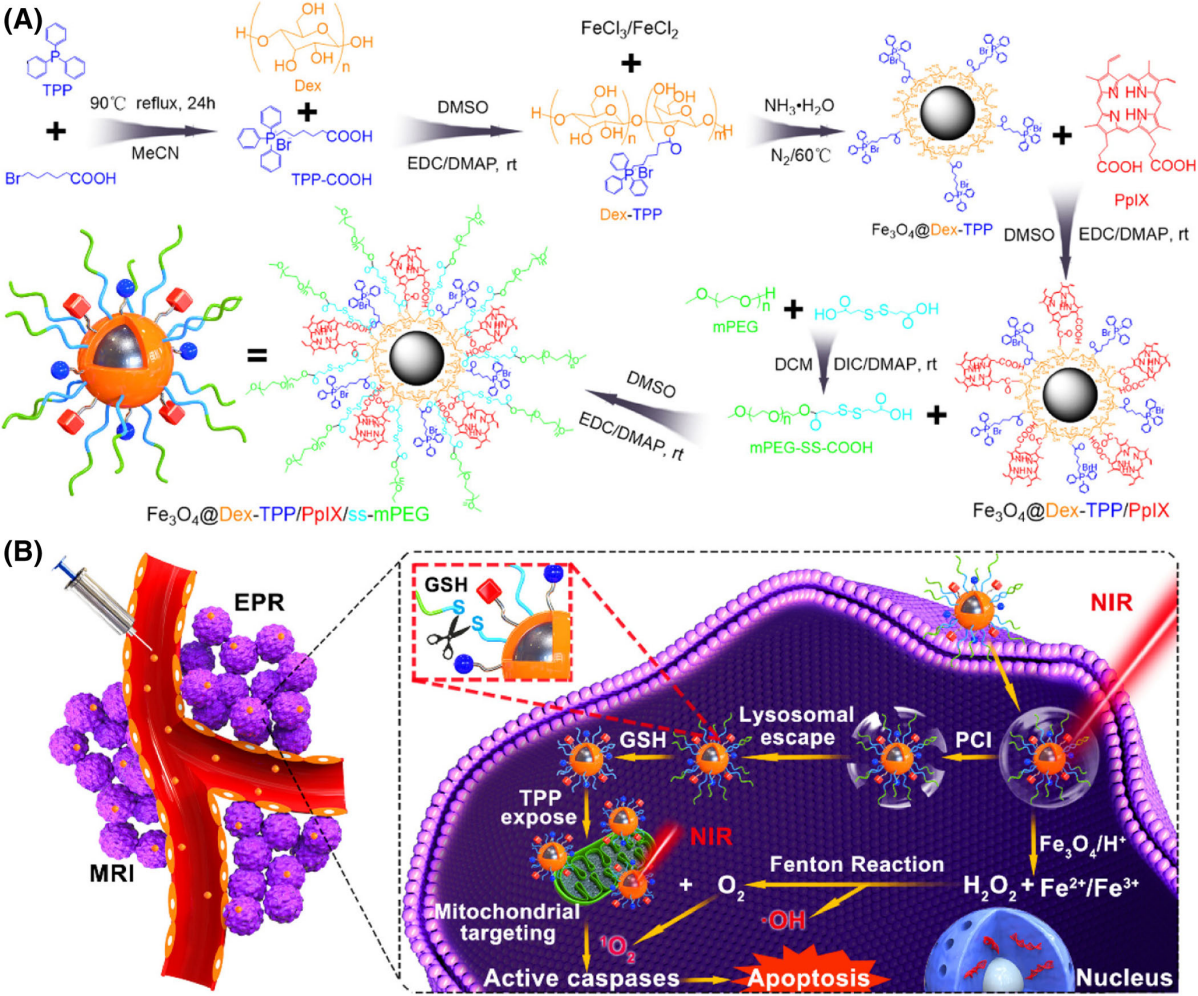
Scheme illustrating of multifunctional modification based on Fe_3_O_4_ nanoparticles and delivery photosensitizer (protoporphyrin IX) for Fenton reaction‐assisted photodynamic therapy of cancer. (A) The preparation route of Fe_3_O_4_@Dex/TPP/PpIX/ss‐mPEG nanoparticles. (B) The Fe_3_O_4_@Dex/TPP/PpIX/ss‐mPEG nanoparticles could target mitochondria and lead mitochondrial apoptosis pathway under laser irradiation for cancer therapy. Reprinted with permission from Ref. [162], Copyright 2019 American Chemical Society.

*Fe_3_O_4_*: Fe_3_O_4_ is a form of iron oxide with low toxicity, high biocompatibility, and magnetic properties that has been approved by the US FDA for biomedicine and widely applied in targeted drug delivery and MRI guidance for cancer.^150^, ^151^, ^152^ Due to the magneto‐thermal energy conversion and delivery ability of Fe_3_O_4_, multifunctional modification strategies based on Fe_3_O_4_ nanoparticles mainly include codelivery, physical coating, and covalent coupling.^153^, ^154^, ^155^, ^156^ Initially, Fe_3_O_4_ nanoparticles could be codelivered with polymeric nanoparticles and be used as the medium of magneto‐thermal energy conversion for magnetic hyperthermia.^157^ For example, Liang et al.^158^ reported a multifunctional bone cement (DOX/Fe_3_O_4_@PMMA), in which DOX and Fe_3_O_4_ nanoparticles were interfused into poly (methyl methacrylate) (PMMA) powders by a mechanical vibration method. These nanoparticles could enhance the release of DOX and realize synergistic magnetic hyperthermia ablation and chemotherapy of osteosarcoma (OS). Based on Fe_3_O_4_ nanoparticles, different polymeric nanoparticles are introduced for multifunctional modification, which could not only increase the stability of Fe_3_O_4_, but also realize the magneto‐thermal energy conversion for cancer therapy. Additionally, Fe_3_O_4_ nanoparticles can be coated with other photothermal polymers, forming a core–shell structure for PTT and magnetic targeting, such as PDA. Moreover, Wang et al.^159^ reported a type of multifunctional core–shell–corona nanohybrid (Fe_3_O_4_@PDA@anti‐miRNA/DNA), in which dopamine was in situ polymerized on the Fe_3_O_4_ NP surface to form the core for PTT to provide magnetic targeting to tumor tissue. They also grafted anti‐miRNA‐21 oligonucleotides (anti‐miRNA) onto its surface stranding as the shell, followed by base pairing with DOX‐conjugated DNA‐8pb corona, which could achieve consumption of tumorigenic miRNA‐21 in tumor cells and the release of DOX–DNA‐8bp for chemotherapy. Moreover, some organic materials (such as oleic acid) have the potential to be used to coat the surface of Fe_3_O_4_ nanoparticles to prevent their aggregation and improve their dispersion.^160^ Simultaneously, these organic materials can introduce various functional groups on the surface of Fe_3_O_4_ nanoparticles for further multifunctional modifications, such as oleic acid and PEG. Ren et al.^161^ developed a multimodified Fe_3_O_4_ nanoplatform (Fe_3_O_4_–PEG–Cy7–EMO), in which the Fe_3_O_4_ coated with oleic acid was conjugated with PEG‐phospholipid and covalently grafted to GFLG–EMO and Cy7–NHS. These nanoparticles were shown to kill pancreatic cancer cells by FI/MRI dual‐mode imaging in combination with chemotherapy. Similarly, Hou et al.^162^ used mPEG‐ss‐COOH to coat Fe_3_O_4_ nanoparticles and a delivery photosensitizer (protoporphyrin IX) for Fenton reaction‐assisted PDT of cancer (Figure 3). Furthermore, the structure of Fe_3_O_4_ nanoparticles can also change the size and specific surface area, forming a mesoporous structure, which can increase the drug loading or dispersion by adsorption. Lu et al.^163^ designed a multifunction nanoparticle based on mesoporous Fe_3_O_4_ (PFP–m–Fe_3_O_4_@PGTTCs). In this case, magnetic mesoporous Fe_3_O_4_ was loaded with PFP by adsorption to form a PFP–m–Fe_3_O_4_ complex, which induced a magnetothermal effect to improve thermal ablation and achieve MRI and ultrasound imaging. Additionally, Deng et al.^164^ used the glypican‐3 (GPC3) peptide to modify mesoporous Fe_3_O_4_ for specifically target hepatocellular carcinoma tumor cells and achieving combined ultrasound/photoacoustic imaging (PAI).

Superparamagnetic iron oxide nanoparticles (SPIONs) are synthesized by decreasing the particle size of Fe_3_O_4_. Due to their large specific area and superparamagnetism, SPIONs are regarded as multifunctional nanoparticles to directly deliver drugs and are widely used in MRI and drug delivery in cancer.^165^, ^166^ For example, Chen et al.^167^ prepared gelatin microspheres dual‐loaded adriamycin (ADM) and Fe_3_O_4_ nanoparticles (ADM/Fe_3_O_4_ MS), in which the ADM/Fe_3_O_4_ MS were obtained using gelatin, ADM hydrochloride, and Fe_3_O_4_ nanoparticles via the high‐voltage electrospray method. The introduction of SPIONs based on Fe_3_O_4_ enabled microspheres to possess the ability to induce hyperthermia and excellent T_2_‐weighted MRI properties.

*Au*: Au nanoparticles consist of small Au particles forming nanoparticles in the shape of nanospheres, nanorods (NRs), nanocones, and nanomushrooms.^168^, ^169^, ^170^, ^171^ Au nanoparticles have a large specific surface area and optical properties that allow drugs or bioactive agents to aggregate on the surface, and are widely used in cancer therapy.^172^, ^173^ Due to the large specific surface area for drug delivery, multifunctional modification strategies based on Au nanoparticles can be chemically or physically conjugated.^174^, ^175^ Initially, Au nanospheres were used to drugs or bioactive agents by noncovalent coupling through disulfide bonds (Au─S bond). Liu et al.^176^ used thiolated multidrug‐resistance protein 1, multidrug resistance‐associated protein 1, and breast cancer resistance protein combined with AuNP via Au─S bonds, forming MBs–AuNP. These nanoparticles were hybridized with three target mRNAs to reduce protein expression and restore the quenching fluorescence for in situ imaging. Au nanoparticles could combine with thiol groups to form the stability Au─S bond, which provide the great potential to prepare multifunctional Au nanoparticles. Similarly, Kim et al.^177^ described a nanoconjugate composed of Au nanosphere (Au nanoparticles) modified by photosensitizers by Au─S bonds for the treatment of glioblastoma multiforme with PTT and PDT. Zhang et al.^178^ also used cysteamine hydrochloride to modify Au nanoparticles using Au─S bonds for a cell fluorescence imaging (FI) probe. Additionally, Au nanospheres elongated along one direction could form Au nanorods (AuNRs), which have the same particle size as Au nanospheres, but a larger specific surface area for enhancing drug loading. Indeed, Pulagam et al.^169^ constructed multifunctional AuNRs, which were grafted with PEG methyl (mPEG) and modified by cobalt (III) bis (dicarbollide) (COSAN) via Au─S bonds. The high density of boron atoms on the AUNR surface enabled them to increase the local temperature under near‐infrared light irradiation and realize the combination therapy of boron neutron capture therapies and PTT. Moreover, multifunctional Au nanoparticles could be modified by other metal materials by electrostatic interaction and used as a biosensor for monitoring cancer therapy, such as MoS_2_. Indeed, Hu et al.^179^ prepared an electrochemical cytokeratin fragment antigen 21‐1 (CYFRA21‐1) biosensor based on Au nanoparticles. To achieve this, they used HCl and LiF as etchant to react with Ti_3_AlC_2_ forming accordion‐like Ti_3_C_2_T*_x_*, and the MoS_2_ was embedded on the surface and crevices of Ti_3_C_2_T*_x_*. The HAuCl_4_ was redox on the surface of MoS_2_ nanosheets forming AuNPs@MoS_2_@Ti_3_C_2_T*_x_*. These prepared nanoparticles were combined with Nafion–Au nanoparticles loaded with Ab1, BSA, and CYFRA21‐1 to obtain a biosensor, which could monitor nonsmall cell lung cancer because of its sensitive detection of CYFRA21‐1.

Au nanoparticles can be combined with Fe_3_O_4_ to form Fe_3_O_4_/Au hybrid nanoparticles, which may have spherical, heterodimer, or core–shell structures.^180^, ^181^, ^182^ Due to the magnetic effect of Fe_3_O_4_ and the optical properties of Au, multifunctional modification strategies based on Fe_3_O_4_/Au hybrid nanoparticles primarily involve coating of the Fe_3_O_4_ surface with an Au forming core–shell structure, followed by covalent conjugation with other agents via the Au─S bond.^183^ The Fe_3_O_4_/Au hybrid nanoparticles are synthesized with a core–shell structure, and the Au surface could be modified by an Au─S bond, such as NHS‐containing agents. Indeed, Rajkumar et al.^184^ synthesized a multifunctional core–shell Fe_3_O_4_@Au hybrid nanoparticle (Fe_3_O_4_@Au–DOX–mPEG/PEG–FA nanoparticles) to load DOX. HAuCl_4_ was reduced at the surface of Fe_3_O_4_ nanoparticles, forming Fe_3_O_4_@Au nanoparticles. l‐Cysteine methyl ester was conjugated to the surface of Fe_3_O_4_@Au nanoparticles by forming an Au─S bond and then connected with NHS–mPEG and NHS–PEG–FA by forming amide bonds to obtain Fe_3_O_4_@Au–mPEG/PEG–FA nanoparticles, which could be used to load DOX for MR/CT imaging and synergistic chemotherapy/PTT for cancer. However, compared with organic nanoparticles, the disadvantages of multifunctional metal nanoparticles (Fe, Au) are the safety of in vivo metabolism, high cost synthesis materinals, and biocompatibility in clinical translation.

*Cu*: Copper (Cu) is a transition metal with excellent thermal and electrical conductivity, which can be used as a heat sink and a high thermal conductivity material.^185^, ^186^ Due to its large specific surface area and strong adsorption capacity for drug delivery, Cu nanoparticles can be used as multifunctional nanoparticles and hybridize with other nanoparticles to form nanocomplexes. According to the form of Cu, there are three main strategies based on the Cu multifunctional nanoplatform.^187^ Initially, due to their adsorption and redox reaction ability, the Cu ion could redox by glutathione (GSH) in cellular and chelate with other elements by forming coordination bonds, such via oxygen and sulfur atoms.^188^, ^189^ Chen et al.^190^ prepared a GSH‐responsive nanoparticle (Cu–IXZ@DSF) based on Cu, in which the Cu was chelated with the ortho‐hydroxy groups of ixazomib (IXZ) and was encapsulated with liposomes to codeliver disulfiram (DSF). The loaded DSF could be reduced to diethyldithiocarbamate by GSH, and then the sulfur atom could compete Cu^2+^ in Cu–IXZ to enhance drug release and tumor chemotherapy. They used the adsorption and redox reaction ability of Cu to modify other bioactive agents, and achieved Cu‐based multifunctional nanoparticles for cancer treatment in GSH response. Additionally, copper sulfide (CuS) nanoparticles have excellent photothermal conversion and can be functionally modified by a Cu─SH bond, which is widely used in tumor combination therapy based on NIR‐responsive PTT.^191^, ^192^ For example, Chen et al.^193^ developed an NIR light‐triggered thermo‐responsive multifunctional nanoparticle based on CuS (CuS–RNP/DOX@PEI) for controlled clustered regularly interspaced short palindromic repeat‐associated Cas9 nuclease (CRISPR–Cas9) ribonucleoprotein delivery. DNA‐SH fragments were combined with CuS nanoparticles by Cu─SH binding and assembled with Cas9 ribonucleoprotein (Cas9 RNP) through the principle of base complementary pairing. The DOX was inserted into the GC‐sites of the DNA‐SH/sgRNA complementary double strand, and finally PEI was coated to obtain CuS RNP/DOX@PEI. The prepared multifunctional nanoparticles could improve the release of Cas RNP under NIR light irradiation and realized photothermal cancer therapy for controllable gene editing. Meanwhile, CuS could be synthesized with a hollow porous nanostructure, which could deliver drugs in the porous channels with high loading by physical absorption. Moreover, Hao et al.^194^ reported using hollow porous CuS nanoparticles to codeliver six drugs for preventing tumor recurrence. The CuS was loaded with oxaliplatin in the hollow core and then modified by FA–polymer, TLR7/8 agonist, mannose, and poly(acetone oxime acrylamide) to induce immunogenic cell death (ICD) along with PTT. Furthermore, copper oxide (CuO) nanoparticles have excellent physical adsorption capacity and codelivery with polymeric nanoparticles, which could be used to enhance cancer therapy with PTT.^195^, ^196^

*TiO_2_*: Titanium (Ti) is a metal element with excellent ductility that is easily oxidized into various oxides in the air, among which, titanium dioxide (TiO_2_) is the most important stable oxide.^197^ TiO_2_ nanoparticles are poorly soluble and have low toxicity, and have been approved by the US FDA as a food color additive.^198^, ^199^, ^200^ TiO_2_ nanoparticles can kill tumor cells through photocatalytic reaction and are widely used as a sensitizer in cancer SDT.^201^, ^202^, ^203^ Due to the high surface area for drug delivery ability, multifunctional design strategies based on TiO_2_ nanoparticles are modified by physical adsorption or chemical covalent coupling. The surface of TiO_2_ nanoparticles could be modified through ionic bonds and electrostatic adsorption, such as Chlorin e6 and immune adjuvant. Indeed, Lin et al.^204^ developed a multifunctional nano sonosensitizer (TiO_2_–Ce6–CpG) based on TiO_2_ for the codelivery of Chlorin e6 and CpG oligonucleotide. The hydrolyzed TiO_2_ nanoparticles were loaded with Ce6 and CpG ODN onto the surface based on ionic bonds and electrostatic adsorption, forming TiO_2_–Ce6–CpG, which inhibited tumor growth in combination with TiO_2_–Ce6‐guided SDT and anti‐PD‐L1 antibody checkpoint blockade. They utilized the advantage of high surface area in TiO_2_ to combine with photosensitizers through physical absorption and delivered immune agonists at the same time, which realized the immune‐PDT for cancer based on multifunctional nanoparticles of TiO_2_. Similarly, Wang et al.^205^ reported the immunogenicity and adjuvanticity of TiO_2_ microparticles decorated with nanospikes to deliver monophosphoryl lipid A for cancer treatment. Additionally, the hydrogen groups on the surfaces of TiO_2_ can adsorb other molecules with hydrogen bonds, such as PEG. For example, Geng et al.^206^ designed multifunctional ultrafine W‐doped TiO_2_ NRs (W‐TiO_2_–PEG) based on TiO_2_ nanostructures. TiCl_4_ and tungsten hexacarbonyl (W(CO)_6_) forming W‐TiO_2_ NRs were generated using a one‐step high‐temperature organic‐phase method and then modified by PEG‐600 by forming hydrogen bonds on the surface to form W‐TiO_2_–PEG NRs. The prepared NRs could improve ROS generation and kill OS cells under SDT–CDT combination therapy.

**FIGURE 4:**
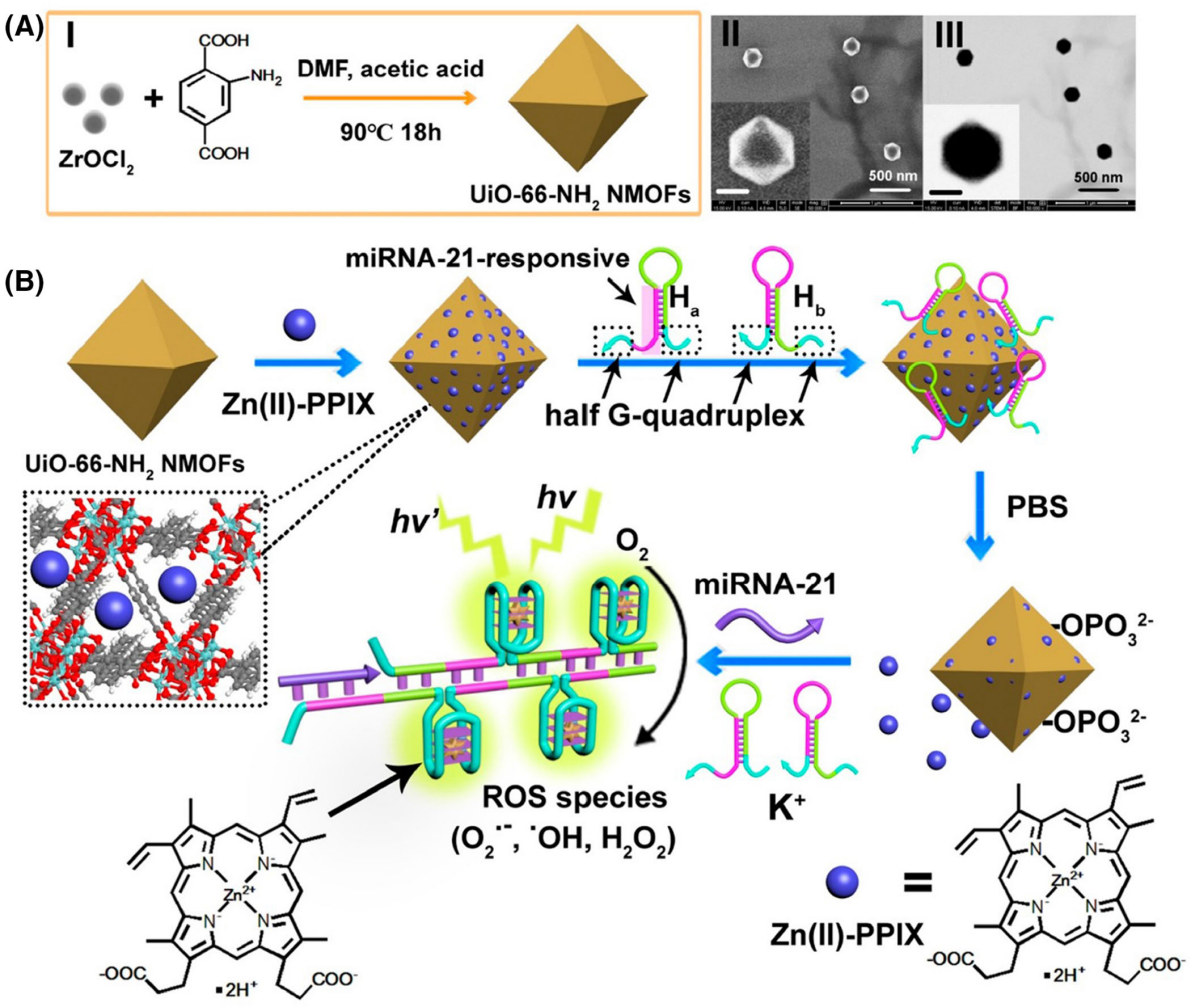
(A) The UiO‐66–NH_2_ NMOFs were synthesized by the reaction of ZrOCl_2_ with aminoterphthalic acid ligand. (B) The UiO‐66–NH_2_ NMOFs loaded with Zn (II)–PPIX, and the nucleic acid hairpins H_a_ and H_b_ were bound to the vacant ligation sites of Zr^4+^‐ions of NMOFs. Reprinted with permission from Ref. [216], Copyright 2022 American Chemical Society.

*MOFs*: Metal‐organic frameworks (MOFs) are compounds formed with metals and organic ligands by organic linkers.^207^ Due to their cage‐like structure, MOF can adsorb numerous drugs in the porous channels to realize codelivery.^208^, ^209^ Functional groups can be introduced in MOFs by different organic ligands, providing additional multifunctional reaction sites for cancer therapy. Due to the drug loading and delivery ability, multifunctional modification strategies based on MOFs include physical absorption and covalent coupling by the functional groups of organic ligands.^210^, ^211^, ^212^, ^213^ According to the type of MOF, UiO‐66 is a zirconium‐based MOF, which was formed by terephthalic acid as the organic framework and connected with zirconium tetrachloride.^214^, ^215^ Due to the pores in UiO‐66, drugs could be loaded in the pores by physical absorption. For example, Zhang et al.^216^ proposed a Zn (II)–PPIX‐loaded nucleic acid‐modified NMOF carrier for miRNA‐guided imaging and PDT of cancer cells (Figure 4). The UiO‐66–NH_2_ NMOFs were synthesized by the reaction of ZrOCl_2_ with aminoterphthalic acid ligand, and then loaded with Zn (II)–PPIX. Subsequently, nucleic acid hairpins H_a_ and H_b_ were bound to the vacant ligation sites of Zr^4+^‐ions of NMOFs to obtain miRNA‐21‐responsive Zn (II)–PPIX‐loaded H_a_/H_b_‐locked NMOFs for selective imaging and PDT of cancer cells. The prepared NMOFs offered the high drug loading for miRNA, while ensured drug delivery and realized selective imaging and PDT treatment of cancer cells based on multifunctional nanoparticles MOFs. Additionally, UiO‐66 could be introduced into functional groups (such as COOH or NH_2_) to conjugate with other agents, such as amine groups, by covalent coupling. Xiong et al.^217^ used 2COOH–UiO‐66 to conjugate with the amine groups of antibody and PEI to coat Au nanoparticles to detect protein biomarkers in the early stages of cancer. Moreover, MOF‐5 is a zinc‐based MOF with an organic framework of terephthalic acid and a central metal of Zn^2+^. MOF‐5 has a large specific surface area and could be substituted with other metal icons, such as Gd. Dai et al.^218^ reported a Ga‐doped multifunctional nanoplatform based on MOF‐5 (Gd–MOF‐5). The terephthalic acid in MOF‐5 was regarded as the ligand to conjugate Gd^3+^ forming Gd–MOF‐5, which could achieve modulating immune stimulation signals and improve the efficacy of cancer immunotherapy. Moreover, MOF‐74 is a MN‐based MOF, which was formed by 2,5‐dihydroxyterephthalate as the organic framework and the central metal with Mn^2+^. MOF‐74 can be modified by metal nanoparticles by a metal─sulfur bond, such as Au─S or Ag─S. Liu et al.^219^ described a MnO@C nanocomposite based on Mn–MOF‐74 for amplifying dual‐signal electrochemical sensing signal of a sensitive cancer biomarker. Moreover, Mn–MOF‐74 was synthesized to obtain MnO@C and conjugated with Au nanoparticles and Ag nanoparticles through Au─S and Ag─S bonds. The prepared nanocomposite prevented the agglomeration of MnO and improved its conductivity with metal modification. However, ZIF8^220^, ^221^, ZIF67^222^, ^223^, ^224^, and MIL100^225^, ^226^ were used as multifunctional nanoplatforms to enhance the treatment effect of cancer with PDT/PTT.

*Pt*: Platinum (Pt) is a transition metal that is commonly used as an alternative to Pt‐based molecules in cancer therapy, such as cisplatin and oxiplatin (Pt^II^).^227^ Pt(IV) can be oxidized to Pt^2+^ under slightly acidic conditions, which can trigger ICD in the context of tumor chemo‐immunotherapy.^228^, ^229^ Due to the presence of empty orbitals in the valence electron shell of Pt, multifunctional modification strategies based on Pt nanoparticles form coordination bonds with other elements.^230^, ^231^, ^232^ Pt nanoparticles can be complexed with nonmetallic elements through coordination bonds, such as Pt‐N_3_. Indeed, Lu et al.^233^ prepared dual‐sensitive dual‐prodrug nanoparticles (DD nanoparticles) based on Pt nanoparticles. The demethylcantharidin (DMC)–Pt (IV)–DMC prodrug was synthesized with DMC, AgNO_3_, and NaN_3_ to form a Pt─N_3_ bond, which was modified by mPEG_2k_─NH_2_ to obtain DDNP nanoparticles. The Pt─N_3_ bond of DD nanoparticles could be broken under UVA light irradiation and stability in the dark, imparting DD nanoparticles with an “on‐off” effect to exert a greater anticancer effect. These modification strategies of Pt combined with other drugs through coordination bonds realized the dual‐sensitive to light and pH of Pt‐based multifunctional nanoparticles for cancer treatment. Additionally, Pt nanoparticles can be incorporated or coated with other polymers through physical absorption, such as PEG. Sun et al.^234^ prepared a multifunctional pH response nanoplatform (Pt@D nanoparticles) based on Pt nanoparticles. The PBA–PEG–PBA copolymer was mixed with H_2_PtCl_6_ to form PBA–Pt, and was BLZ‐945 mixed with PLGA to form PNP. The PNP was modified by dextran, forming D nanoparticles, and covalent with PBA–Pt by a pH‐responsive boronate bond to obtain Pt@D nanoparticles. The boronate bond could be broken under an acidic pH to release drugs to improve tumor cell killing in cancer chemo‐immunotherapy. Moreover, Pt can be synthesized with a mesoporous structure to form mesoporous Pt nanoparticles, which possess the properties of large specific surface areas and adjustable apertures for enhancing high drug loading through electrostatic adsorption. Fu et al.^235^ reported a multifunctional nanoparticle (PEG@Pt/DOX) based on mesoporous Pt (mesoPt) nanoparticles. The mesoPt was synthesized using pluronic F127 surfactant as a structure‐directing agent, and modified by SH–PEG–COOH on the surface, forming PEG@Pt nanoparticles. PEG@Pt was loaded with DOX by electrostatic adsorption forming PEG@Pt/DOX with high drug loading. Due to the Pt nanoparticles, PEG@Pt/DOX exhibited a strong photothermal transformation ability and CT imaging‐guided PTT.

*MoS_2_*: Molybdenum disulfide (MoS_2_) is a two‐dimensional transition metal dichalcogenide composed of molybdenum and sulfur.^236^, ^237^ MoS_2_ has a layered structure, in which the molybdenum atomic layer is sandwiched by the sulfide ion layer through van der Waals forces, forming a three‐layer structure.^238^ Due to the sulfur atom being exposed on the surface of MoS_2_ nanosheets, forming defect vacancies, the multifunctional design strategies based on MoS_2_ nanosheets mainly include covalent or noncovalent combinations with other metals and organic small molecules by strong absorption.^239^, ^240^ In detail, the defect vacancies of MoS_2_ nanosheets can form a coordination bond with thiol, forming S─S bonds to enhance drug release with a GSH response.^241^ Liu et al.^242^ prepared a multifunctional nanoparticle (MoS_2_–SS–HA–CPT) based on MoS_2_, in which the MoS_2_ was bound with HA–SH though a disulfide bond, forming MoS_2_–SS–HA. Due to the modification of the disulfide bond, MoS_2_–SS–HA exhibited redox stimuli‐responsiveness in the GSH environment. MoS_2_–SS–HA showed strong NIR absorbance and the capability of photothermal conversion. After loading with camptothecin (CPT), MoS_2_–SS–HA–CPT could target tumor cells for the synergetic chemo‐PTT for cancer. They utilized the property of MoS_2_ to couple with thiol through forming S─S bonds and realized the redox/NIR dual response for cancer therapy based on multifunctional MoS_2_ nanoparticles. Additionally, the defect vacancies of MoS_2_ nanosheets can be encapsulated by polymers (such as HA, PEG, PEI, and CS), forming a C─S bond, which can enhance the drug release and be used in combination with photothermal and chemotherapy for cancer therapy.^243^, ^244^, ^245^ Dong et al.^246^ prepared MoS_2_–PEI–HA based on MoS_2_ nanoparticles. MoS_2_ nanosheets were coated with HA to introduce NH_2_ and COOH groups. The nanosheets could release drugs under acidic conditions, NIR laser lighting, and the enzyme HAase. Following conjugation with NOTA, the nanocomposite could chelate with ^64^Cu for PET imaging. After loading with DOX, MoS_2_–PEI–HA–DOX could release drugs in the tumor acidic microenvironment and be guided by PET imaging for cancer chemotherapy. Similarly, Liu et al.^247^ reported an MoS_2_ composite nanosheet material BBPL–MoS_2_–LP, in which the lipoic acid (LA)–PEG and LA–PEI‐modified MoS_2_ was combined with biotin–BSA through amide bonds and loaded DOX to achieve synergistic targeted chemo‐PTT.

*Mn*: Manganese (Mn) is a transition metal that is easily oxidized, with oxidation products including MnO, Mn_2_O_3_, MnO_2_, and Mn_3_O_4_. As Mn is a cofactor for many metabolic enzymes and has efficient redox properties, it can decompose H_2_O_2_ in tumors and generate highly toxic ROS, causing oxidative damage to tumor cells.^248^, ^249^ Due to their biocatalytic and redox abilities, multifunctional modification strategies based on Mn‐based nanoparticles are modified by other agents by electrostatic attraction to form nanocomplexes.^250^, ^251^, ^252^ For example, Mn ions could be stored in oil phase and coated with pH‐sensitive liposome to form a core–shell structure. Zhu et al.^253^ developed a multifunctional nanosystem (NanoMn–GOx–PTX) based on MN for the codelivery of the glucose oxidase (GOx), PTX. Na_2_HPO_4_ solution (mixed with GOx) was added to the configured oil phase and MnCl_2_ and DOPA were added to another oil phase, forming a monolayer lipid NanoMn–GOx core. PTX was loaded into a phospholipid bilayer shell to obtain NanoMn–GOx–PTX. These nanoparticles could generate ROS and enhance the anticancer effect. Additionally, Mn ions could be modified by CO_3_^2−^ ions by electrostatic attraction, forming MN ion carbonate with pH responsive abilities. Qi et al.^254^ constructed multifunctional nanotheranostics (BMC nanoparticles) based on pH‐responsive MnCO_3_ nanocarriers to load DOX. They synthesized poly(ethylene glycol)‐*b*‐poly(l‐aspartic acid) to conjugate with Mn^2+^ ions via electrostatic attraction and modified the complex with CO_3_^2−^ ions through an in situ mineralization approach to form biomineralized MnCO_3_ (BMC) nanoparticles. After loading DOX, the prepared nanoparticles effectively killed tumor cells by a chemodynamic therapy (CDT)/chemotherapy combination. Moreover, Mn ions could be redoxed by KMnO_4_, forming MnO_2_, which could be modified by polymers, such as polypyrrole. Li et al.^255^ reported multifunctional core‐sheet polypyrrole@MnO_2_ nanocomposites (PPy@MnO_2_) to load methylene blue (MB). PPy@MnO_2_ was obtained using an in situ redox reaction between PPy and KMnO_4_. The mPEG–NH_2_ was conjugated on its surface by electrostatic attraction and then encapsulated MB through charge–charge attraction, forming PPy@MnO_2_–PEG–MB. The obtained nanocomposites showed high tumor inhibition effects under combined PPy‐mediated PTT and PDT. Moreover, mesoporous MN trioxide (Mn_2_O_3_),^256^ MN phthalocyanine (MnPc),^257^ Mn_3_O_4_ nanoparticles,^70^, ^258^ and MnO*_x_* component^259^ were prepared based on Mn‐based nanoparticles for enhancing cancer therapy with MRI, PTT, and SDT.

*Bi_2_S_3_*: Bismuth sulfide (Bi_2_S_3_) is an inorganic semiconductor material and is the main component of bismuthinite.^260^, ^261^ Due to its excellent biocompatibility and imaging performance, Bi_2_S_3_ was used as an imaging contrast agent and PTT agent in cancer therapy.^262^, ^263^ Regarding the imaging and delivery ability of Bi_2_S_3_, multifunctional design strategies based on Bi_2_S_3_ nanoparticles include codelivery with drugs or other agents to enhance drug delivery and cancer therapy with imaging guidance.^264^, ^265^, ^266^, ^267^ Initially, multifunctional Bi_2_S_3_ nanoparticles could be coated on proteins by biomineralization and codelivery with metal nanoparticles,^268^ such as Au atoms. Nosrati et al.^269^ prepared a type of bimetallic theranostic nanoparticle, Bi_2_S_3_@BSA–Au–BSA–MTX–Cur, in which they used BSA to Bi_2_S_3_ nanoparticles by biomineralization and modified them with Au nanoparticles to deliver methotrexate (MTX) and Cur. The photothermal properties of Bi_2_S_3_ nanomaterials could be improved by incorporating Au atoms. Indeed, MTX conjugated to Bi_2_S_3_–Au nanoparticles as a targeted chemotherapeutic drug, and the loaded CUR could be used for both radiosensitizing and radioprotection. This platform served as a contrast agent, drug carrier, and nanoradiosensitizer, demonstrating effective anticancer effects. Additionally, multifunctional Bi_2_S_3_ nanoparticles could be capsulated by polymeric nanoparticles to realize codelivery, such as PLGA. Zhao et al.^270^ developed a multifunctional nanoparticle with targeted therapeutic function and dual‐modal imaging guidance based on Bi_2_S_3_ for treating ovarian cancer. The Bi_2_S_3_ nanoparticles were encapsulated with PLGA–PEG–FA to deliver DOX and PFP, forming FBPD nanoparticles. These nanoparticles showed a good therapeutic effect of PTT combined with chemotherapy under the guidance of CT and PA imaging. Therefore, Bi_2_S_3_ nanoparticles could codeliver with multiple organic or inorganic nanoparticles to construct multifunctional nanoparticles, which realized imaging‐guided photodynamic cancer therapy.

#### 2.2.2 Nonmetallic nanoparticles

*MSNs*: Mesoporous silica nanoparticles (MSNs) are composed of the mesoporous form of silica with a pore size of 2–50 nm.^271^, ^272^ MSNs contain numerous silanol groups on the surface, which could be hydrolyzed under an acidic or alkaline environment, while silanol groups could be modified by various organic functional groups through covalent bonds or electrostatic interactions.^273^, ^274^ Drugs could be adsorbed in the pore channels of MSNs, which can protect drugs from being degraded and increase the solubility of drugs for cancer therapy.^275^ Based on the drug delivery ability of MSNs, the multifunctional design strategies of MSNs include coating with other compounds by electrostatic interactions or modifying with silanol groups by covalent coupling. Multifunctional MSNs could be coated with metal or organic materials by electrostatic interactions, such as MoS_2_, PDA, ICG, and CS.^276^ Wu et al.^277^ prepared a multifunctional PMOs–DOX@MoS_2_–PEI–BSA–FA complex based on periodic mesoporous organosilica nanoparticles (PMOs). PMOs were coated with MoS_2_ by electrostatic interactions and weak thiol reactions and modified by PEI–BSA–FA to deliver DOX to enhance drug release under laser irradiation to achieve synergistic chemo‐photothermal targeted therapy. Similarly, Zhang et al.^278^ used PDA to coat mesoporous silica (MSNs) and modified it with ICG and RGD to absorb ammonium bicarbonate and DOX in the pores for combining cancer therapy with PTT/PDT. Park et al.^279^ also constructed multifunctional nanostructures (MONA) based on a mesoporous‐silica nanosphere coated on an anti‐EpCAM grafted Au layer for multifunctional cellular targeting and light‐driven delivery of DOX to breast cancer. These modification strategies of coating realized cellular targeting and photodynamic cancer therapy based on multifunctional MSNs nanoparticles. Additionally, the silanol groups on the surface of MSNs could be modified by various organic functional groups through covalent bonds, such as NH_2_ and COOH.^280^, ^281^ Wagner et al.^282^ reported multifunctional nanocarriers (MSN–Ph_in_–avidin_out_) based on core–shell MSNs. The core of the MSNs was functionalized with phenyl to accommodate hydrophobic R848, and the outer surface was functionalized with carboxyl groups to allow continuous modification to attach a pH‐responsive acetal linker and a biotin‐avidin complex for enhancing anticancer effects with immune reaction. Similarly, Yang et al.^283^ reported that carboxylated dendritic MSNs could be modified by PEI by forming amide groups and loaded with microRNA‐125a for cancer immunotherapy. Lohiya et al.^284^ also conjugated carboxylated CS with the hydroxyl group on mesoporous silica to deliver DOX for targeted therapy for breast cancer.

Hollow MSNs (HMSNs) are a derivative of MSNs with the dual advantages of being mesoporous and hollow.^285^ The regular pore structure and spherical hollow part increases the specific surface area and significantly improves the drug delivery ability.^286^ HMSNs could encapsulate hydrophilic and hydrophobic drugs, up to three drugs, which combine chemotherapy with various antitumor strategies.^287^ Due to the drug delivery of HMSNs, multifunctional modification strategies based on HMSNs include codelivery with multiple drugs by electrostatic adsorption or modification by silanol groups. For example, Goel et al.^288^ designed CuS‐coated and [^89^Zr]‐labeled hollow mesoporous silica nanoshell nanoparticles ([^89^Zr]CSNCs–PEG_10k_) to load porphyrin molecules (TCPP). Negatively charged CuS–Cit was grafted onto the positively charged HMSN–NH_2_ by electrostatic adsorption and conjugated branched PEG via a condensation reaction, encapsulating TCPP to form [^89^Zr]CSNCexosomes–PEG_10k_. The prepared nanoparticles could produce excellent anticancer effects under CuS‐guided PTT and TCPP‐mediated PDT.

*Carbon‐based nanoparticles*: Carbon‐based nanoparticles consist of carbon elements, mainly including graphene, graphene oxide (GO), carbon quantum dots (CQDs, C‐dots or CDs), and carbon nanotubes.^289^ Carbon‐based nanoparticles have a large specific surface area, strong drug absorption, and easy functional modification, and are commonly used for drug delivery.^290^ Due to the drug delivery ability of carbon nanoparticles, multifunctional modification strategy‐based carbon‐based nanoparticles can be covalently modified and physically absorbed.^291^, ^292^, ^293^, ^294^, ^295^, ^296^ According to the different preparation and structure from carbon nanoparticles, graphene is a single layer formed by the close packing of sp^2^‐bonded carbon atoms, and its diameter is the size of a single carbon atom. Due to the property of photothermal conversion under NIR, multifunctional construct strategies of graphene include drugs loaded through π–π stacking and coordinated with other metals by intercalating in the carbon structure.^297^, ^298^ For example, Chen et al.^299^ reported a Gd@GCNs nanoparticle based on gadolinium‐encapsulated graphene carbon, in which Gd acted as a coordination center in the formation of crystalline carbon layers, which intercalated in the final carbon structure. Gd@GCNs showed strong fluorescence and high T_1_ relaxivity as a dual‐modal imaging probe for cancer therapy.

GO is synthesized by the oxidation of graphite with a two‐dimensional honeycomb network structure. Functional groups can be introduced in the synthesis of GO, and include hydroxyl groups, carboxyl groups, epoxy groups, and carbonyl groups. Due to the functional groups in GO, multifunctional modification strategies of GO are mainly via covalent coupling. For example, the epoxy groups of GO could be conjugated with amine groups by a ring‐opening nucleophilic addition reaction.^300^, ^301^ Zeng et al.^302^ described a multifunctional nanocomposite based on GO for OS. The epoxy groups of GO were conjugated with the amine groups of PEG by a ring‐opening nucleophilic addition reaction to load with ICG to enhance PDT/PTT and inhibit ATP production to induce tumor apoptosis. Additionally, the carboxyl groups in GO are modified by bioactive agents containing amine groups, forming an amide bond. Xing et al.^303^ prepared a multifunctional nanodrug GDYO–CDDP/DOX@DSPE–PEG–MTX (GCDM) based on graphdiyne oxide (GDYO) for targeted cancer photo‐chemo synergetic therapy. They developed a three‐dimensional framework GDYO–CDDP using the amide reaction of GDYO and cisplatin (CDDP). GCDM nanoparticles were then obtained using DSPE–PEG2000–MTX and GDYO–CDDP by self‐assembly. The prepared nanoparticles could load DOX through π‐π stacking and treatment cancer with photo‐chemo synergetic therapy.

CQDs (C‐dots, or CDs) are nanomaterials composed of carbon, hydrogen, oxygen, and nitrogen, with a particle size <10 nm.^304^, ^305^, ^306^, ^307^, ^308^ CDs not only possess excellent photostability and efficient catalytic ability but also have surface modification flexibility. Based on the fluorescence ability of CDs, multifunctional CDs are covalently modified the free amine and hydroxyl groups.^309^ In detail, the metal cations can be adsorbed on their surface by conjugating with amino and hydroxyl groups and cause fluorescence quenching of CDs. With the reduction reaction of metal cations, the fluorescence of CDs could be realized by FI. For example, Li et al.^310^ developed a TME stimuli‐responsive nanomedicine (Fe–CD) with CDs and Fe (III). The amino and hydroxyl groups on the surface of the CDs were conjugated with Fe (III). The Fe was redoxed by GSH in the cellular and restored the strong fluorescence of CD, which could achieve FI‐guided CDT. Additionally, CDs could be modified by magnet metals and modified by polymers through hydrophobic interactions. Jia et al.^311^ prepared a magnetofluorescent carbon dot (Mn–CD) based on MN (II) phthalocyanine (Mn–Pc). The Mn–CD prepared by Mn–Pc through a solvothermal method and modified by DSPE–PEG. The results showed that Mn–CD had efficacy as an H_2_O_2_‐driven oxygenerator for bimodal fluorescence (FL)/MRI‐guided PDT. However, compared with organic nanoparticles, multifunctional carbon‐based nanoparticles are still poor in hydrophobicity, high cost in synthesis, and small production.

*Black phosphorous*: Black phosphorous nanosheets are exfoliated from black phosphorus (BP) with a flaky monolayer structure; as a result, black phosphorous nanosheets have large specific surface area, which could adsorb drugs with high drug loading capacity.^312^, ^313^ Black phosphorous nanosheets are widely used in cancer therapy with excellent photothermal conversion ability for PTT/PDT/SDT.^314^ Based on the drug delivery ability, multifunctional design strategies based on black phosphorous nanosheets include codelivery with other agents by electrostatic adsorption to enhance drug release with PTT/PDT/SDT.^315^ Black phosphorous nanosheets can be codelivered with polymeric nanoparticles by electrostatic adsorption, such as PAMAM and PEG. For example, Peng et al. constructed a multifunctional nanoplatform based on black phosphorous nanosheets (BP NS–PAMAM@DOX–HA) to deliver DOX.^316^ The PAMAM was grafted onto the surface of BP NSs by electrostatic adsorption and coupled with HA to deliver DOX, which could produce excellent anticancer effects under chemo‐photothermal synergistic cancer therapy. Based on the large specific surface area of, black phosphorous nanosheets multifunctional modified with polymeric nanoparticles, and realized NIR thermal imaging‐guided chemo‐photothermal cancer therapy. Similarly, Zhang et al.^317^ also designed mPEGylated black phosphorous nanoparticles (BP) modified by HA through a condensation reaction to form HA–BP for targeted and multiple combination therapies of cancer. Additionally, black phosphorous nanosheets could be mixed with other metal materials by charge coupling and electrostatic adsorption, such as Zn and Fe. Kang et al.^318^ described a composite nanosheet (M‐RP/BP@ZnFe_2_O_4_) based on red/BP (RP/BP). The ZnFe_2_O_4_ nanoparticles were synthesized in situ on the RP/BP NSs surface by electrostatic adsorption. The combination of BP and RP achieved photocatalytic water splitting to generate H_2_O_2_ and O_2_. ZnFe_2_O_4_ could promote PDT by catalyzing the Fenton reaction and regulating the intracellular redox level through GSH consumption. Similarly, Li et al.^319^ used Zn^2+^ to modify BP by charge coupling to deliver DOX for immunotherapy and PTT.

### 2.3 Biomimetic backbone

#### 2.3.1 Cell membrane biomimetic nanoparticles

Cell membrane biomimetic nanoparticles are an emerging nanobiomimetic technology formed by encapsulating nanoparticles or drugs with the membranes of various cells.^320^ The main component of cell membranes are phospholipids with a bilayer structure, which can prolong the circulation time in the body and achieve homologous targeting delivery for cancer therapy.^321^, ^322^ Cell membranes can be coated on the drugs or other nanoparticles through a physical coextrusion approach, sonication, and microfluidic electroporation forming a core–shell structure.^323^ Due to the target delivery of cell membrane nanoparticles, multifunctional modification strategies based on cell membrane biomimetic nanoparticles include noncovalent coating and chemical modification.^324^, ^325^, ^326^ Due to the biocompatibility of the cell membrane, the cell membrane can be coated on the photothermal materials to form a core–shell structure, such as Pt, Au,^327^ IR780,^328^ and IR820^329^. For example, Zhai et al.^330^ prepared a traceable bioinspired nanovesicle (MPV) based on a cell membrane‐derived shell. They synthesized gelatin nanogels (NGs) using a two‐step desolvation method and loaded Pt and MB to form MPNGs, and then coated them with red blood cell membranes. The prepared bioinspired nanovesicles could be used in the treatment of 4T1 breast cancer with chemo‐PDT, while the tumor cells could be monitored by multimodal imaging. The prepared MPV multifunctional nanoparticles realized the potential of cell membrane biomimetic nanoparticles for photodynamic cancer therapy. Additionally, the surface of cell membranes of biomimetic nanoparticles could be modified by peptides, such as angiopep‐2. Indeed, Zou et al.^331^ described a multifunctional biomimetic nanomedicine Ang–RBCm@NM–(DOX/Lex) based on functionalized red blood cell membranes (RBCms). The RBCms were modified by angiopep‐2 to encapsulate with a­dextran/DOX/lexiscan nanoparticle to enhance drug release by traversing the BBB and targeting glioblastoma multiforme. Additionally, the cell membranes can be functional modified with other groups due to the amine groups, thiol groups, and carboxyl groups existent on the surface of membrane proteins. For example, Yu et al.^332^ created ^89^Zr‐labeled multicompartment membrane‐derived liposomes (^89^Zr–Df–MCLs), which were prepared by a membrane reassembling method that incorporated Tween‐80 into the 4T1 cell membranes. *p*‐SCN‐deferoxamine conjugated with amine groups of proteins in the bilayer and then radiolabeled ^89^Zr, forming ^89^Zr–Df–MCLs, which effectively killed cancer cells by effective PDT.

Moreover, the hybrid cell membrane is also a multifunctional cell membrane biomimetic nanoparticle strategy. A hybrid cell membrane is prepared by fusing various cell membranes through electrostatic interactions, which contain various surface membrane protein markers from different cells. Based on the delivery capability of a cell membrane, multifunctional hybrid cell membrane nanoparticles are codelivered with functional materials by electrostatic interaction.^333^, ^334^, ^335^ Initially, the cancer cell membrane could be fused with the bacteria membrane by electrostatic interaction to encapsulate photothermal nanoparticle. For example, Guo et al. developed a multifunctional nanoparticle Fe_3_O_4_/MnO_2_/MM/EM/D based on the MCF‐7 breast cancer cell membrane (MM) and *Escherichia coli* membrane (EM).^336^ MM and EM were obtained using ultrasonic and gradient centrifugation with electrostatic interactions and encapsulated with Fe_3_O_4_/MnO_2_ nanoparticles, forming a core–shell structure. These nanoparticles possessed outstanding photothermal properties to achieve PTT and CDT. Additionally, the cancer cell membrane could be fused with the immune cell membrane using a physical ultrasound method. Hao et al. prepared a hybrid membrane‐coated nanosuspensions (DNS‐[C6&DC]m) based on a C6 cancer cell membrane and DC membrane to deliver drug and antigen for antiglioma therapy due to the high drug carrying capacity of nanosuspensions and the professional antigen presentation ability of DC membranes.^337^ Moreover, others used a red blood membrane hybrid with MCF‐7 breast cancer cell membrane to deliver melanin nanoparticles for enhancing cancer therapy with PDT.^334^

#### 2.3.2 Exosomesc

Exosomes, another type of biomimetic nanoparticle, are membranous vesicles actively secreted by cells with a double‐layered membrane structure.^338^, ^339^ Because of stability and transmembrane transport properties, exosomes are used as delivery vectors for virous cancer therapy.^340^, ^341^ Based on the delivery ability of exosome, multifunctional modification strategies based on exosome are noncovalently and covalently modification. Given their high biocompatibility with lipids, exosomes can be codelivered with other functional nanoparticles, such as Fe_3_O_4_. For example, Pan et al.^342^ designed a multifunctional nanosystem (EXO–PMA/Fe–HSA@DOX) based on urinary exosomes for the delivery of DOX. HSA and DOX were conjugated to poly (isobutylene‐alt‐maleic anhydride)‐modified Fe_3_O_4_ and encapsulated into urinary exosomes via electroporation method. Due to the homologous targeting, these nanoparticles could accumulate in prostate cancer cells, providing significant anticancer effects under chemo/CDT. They utilized exosomes multifunctional modified with Fe_3_O_4_, which realized the tumor‐specific targeting and immune escape of exosomes and chemodynamic cancer therapy. Due to the protein in exosomes, there are many amine groups, thiol groups, and carboxyl groups that can be covalently modified by other functional groups, such as amino groups. Qian et al.^343^ designed multifunctional nanoparticles (^131^I‐EM@ALA) based on ^131^I‐labeled exosome mimetic (EM) to deliver 5‐aminolevulinic acid (ALA). 4T_1_ cancer cell membranes squeezed together and modified by the amine groups in ALA to obtain EM@ALA.^131^I was radiolabeled EM@ALA by the chloramine T method, forming ^131^I‐EM@ALA. The prepared nanoparticles produced ROS and killed tumor cells by inducing DNA damage and activating the lysosomal mitochondrial pathway. Additionally, other scholars used cell‐penetrating peptide or magnet nanoparticles to modify exosomes in order to enhance their tumor targeting or dual‐modal detection abilities.^344^, ^345^ However, compared with cationic nanoparticles, multifunctional biomimetic nanoparticles (cell membranes and exosomes) are poor in transfection efficiency, isolation, and purification process.

## 3 APPLICATION STRATEGIES OF MULTIFUNCTIONAL NANOPARTICLES

Due to the variability and strong adaptability of cancer cells, they could adjust their structure or cell properties to adapt to the surrounding environment and continue to survive, which undoubtedly brings huge obstacles to treatment. However, a single functional nanoparticle drug delivery system seems not to be sufficient for diversified tumor treatment strategies. In order to monitor and treat the occurrence and development of tumor cells, multifunctional nanoparticles integrate different functions to further expand the carrier's application, thus achieving two or more capacities. Here, we highlight the delivery, therapy, and imaging function of multifunctional nanoparticles. The summary of functions associated with multifunction nanoparticles are in Table 2.

**TABLE 2:** Summary of functions associated with multifunction nanoparticles

Introduced function	Category	Functional groups/bonds/agents	Application	References
Delivery	Target	Folic acid	Chemotherapy with epithelial carcinoma tumor targeting	^127^
		cRGD	Chemotherapy with nonsmall cell lung cancer targeting	^125^
		Tumor cell membranes	Chemotherapy and immune stimulation with glioma targeting	^337^
		Mucin‐1 aptamer and T22‐NLS peptide	Gene editing with tumor targeting	^360^
		Cell‐type‐specific aptamer and TAT peptide	Targeting gene editing and photothermal therapy	^361^
		AS1411 aptamer and TAT peptide	Gene editing with tumor targeting	^362^
	pH sensitive	Imidazole	Chemotherapy with pH‐sensitive promoting cell uptake	^372^
		Hydrazone bond	Chemotherapy with pH‐sensitive promoting drug release	^109^
		Amide bond	Chemotherapy with pH‐sensitive promoting drug release	^34^
		Zinc–ligand bonds	Cancer therapy with pH‐sensitive enhancing mRNA delivery	^373^
		Cyclic benzylidene acetal	Cancer therapy with pH‐sensitive enhancing mRNA delivery	^374^
		Added anilines to tune the p*K*a	Cancer therapy with pH‐sensitive enhancing mRNA delivery	^375^
	Redox sensitive	Diselenide bond	Protein therapy with redox‐sensitive promoting drug release	^359^
		Disulfide bond	Gene therapy and chemotherapy with redox‐sensitive promoting drug release	^80^
		Thioketals bond	Chemotherapy with redox‐sensitive promoting drug release	^387^
Therapy	Immunotherapy	Toll‐like receptor 7/8 agonist	pH‐sensitive promoting drug release and immune stimulation	^282^
		PD‐L1 inhibitory peptide	MRI‐guided tumor targeting and immune stimulation	^324^
		Ovalbumin	PTT collaborated with immune stimulation	^397^
	Photodynamic (PDT)	IR780	PDT collaborated with chemotherapy	^325^
		Quantum dots‐chlorin e6	PDT collaborated with cell therapy	^409^
		Pyropheophorbide a	PDT collaborated with cancer therapy	^65^
	Photothermal (PTT)	PDA	PTT collaborated with gene therapy	^42^
		Au	PTT collaborated with boron neutron capture therapy	^169^
		Indocyanine green (ICG)	PTT, PDT, and starvation therapy	^419^
	Sonodynamic (SDT)	AIPH	PAI/FI/US imaging‐guided SDT	^431^
		Hematoporphyrin monomethyl ether	PAI‐guided SDT	^428^
		TiO_2_	SDT collaborated with immune stimulation	^429^
	Chemodynamic (CDT)	Fe_3_O_4_	MRI‐guided CDT and immune stimulation	^440^
		MnO_2_	MRI‐guided CDT and chemotherapy	^251^
		Cu	CDT collaborated with chemotherapy	^441^
	PDT/PTT	Au/Chlorin e6	PDT collaborated with PTT	^177^
		Black phosphorus	FI/PAI‐guided PDT, PTT and immune stimulation	^317^
	PDT/CDT	Chlorin e6/hemin	PDT, CDT and ferroptosis	^451^
	PTT/CDT	Cu/black phosphorus quantum dots	PTT collaborated with CDT	^188^
Therapy	CDT/PTT/PDT	MnO_2_/ICG/Au	MRI/FI/thermal imaging‐guided PDT, CDT and PTT	^458^
Imaging	Magnetic resonance imaging (MRI)	SPIONs	MRI‐guided hyperthermia and chemotherapy	^26^
		Zn*_x_* Fe_3‐_ *_x_* O_4_ NPs	MRI‐guided immune stimulation	^468^
		Mn^2+^	MRI‐guided PDT and immune stimulation	^470^
	Fluorescence imaging (FI)	NCBS	FI‐guided ROS‐sensitive drug delivery	^478^
		Cr^3+^/Sn^4+^	FI‐guided chemotherapy	^479^
		Fluorescent cyclic peptide nanoparticles	FI‐guided chemotherapy	^142^
	Photoacoustic imaging (PAI)	Black phosphorus quantum dots	PAI‐guided CDT and PTT	^188^
		PDA	PAI‐guided PTT and chemotherapy	^486^
		Molybdenum	PAI‐guided CDT and PTT	^487^
	X‐ray computerized tomography (CT) imaging	Cu_3_BiS_3_	MRI /CT imaging‐guided PTT and PDT	^445^
		Bi_2_S_3_	PAI/CT imaging‐guided PTT and chemotherapy	^270^
		I_2_	PAI/CT imaging‐guided PTT	^492^
	Multimodal imaging	Fe_3_O_4_/MnO_2_/Ce6	MRI/FI/PAI‐guided chemotherapy and PDT	^497^
		Fe_3_O_4_/perfluoropentane	MRI/PAI/US imaging‐guided hyperthermia	^498^
		^68^Ga/Gd^3+^/Cr^3+^	FI/MRI/PET imaging‐guided chemotherapy and PDT	^499^

AIPH, Zr‐MOF/2,2‐azobis[2‐(2‐imidazolin‐2‐yl) propane] dihydrochloride; NCBS, silicon 2,3‐naphthalocyanine bis(trihexylsilyloxide).

### 3.1 Multifunction in drug delivery

#### 3.1.1 Targeted delivery

Targeting drug delivery systems refer to the selective delivery of drugs to target tissues or target organs by vectors and the mechanism of target delivery mainly includes passive targeting and active targeting.^346^ The advantage of targeted delivery is that the drug can be delivered to the specific treatment site to enhance the drug efficacy, reduce side effects, and achieve the purpose of effective cancer therapy.^347^, ^348^ To realize targeted drug delivery, multifunctional nanoparticles are modified by target function to enhance the activity of the drug at the target site and reducing the toxic and side effects at the nontarget site.^349^, ^350^, ^351^ One of the commonly target modifications is a small molecule compound, such as FA. FA is an essential substance in the process of tumor proliferation, and the folate receptor is highly expressed in most tumor cells as a mediator for tumor cells to absorb FA.^352^, ^353^ Liu et al.^127^ designed a multifunctional nanoplatform (FOBD) using FA‐modified *o*‐nitro‐benzyl ester lipid for delivery of DOX‐targeted therapy in cancer. Due to the high affinity of FA to folate receptors (FRs), the prepared nanoparticles not only achieved liposomal ‐based passive targeting (EPR) delivery, but also actively delivered DOX to tumor cells. This resulted in the enrichment of tumor cell activity after injection into tumor mouse models, achieving significant inhibition of tumor growth. Due to the high expression of integrin receptors on tumor cell membranes, cell penetrating peptides with RGD sequences (Arg–Gly–Asp) can bind to integrin receptors with high affinity, which is beneficial for nanoparticle tumor targeting.^354^, ^355^, ^356^ Gu et al.^125^ prepared a type of targeted ligand c(RGDfC)‐modified multifunctional nanoparticle (CP@NP–cRGD) to deliver CQ and selective FGFR1 inhibitor for targeted therapy for nonsmall cell lung cancer. These nanoparticles could deliver drugs to tumor tissues with high delivery efficiency, and actively target tumor cells through cRGD peptides on the shell. Their results demonstrated that cRGD‐nanoparticles showed more targeted distribution in vivo and effectively prevented the degradation of drugs during the circulation process to maximize the anticancer effect. Additionally, multifunctional nanoparticles coated with tumor cell membranes have the similar antigens and compositions to tumor cells, which show excellent tumor homologous targeting ability.^357^, ^358^ Hao et al.^337^ designed a hybrid membrane coated nanodelivery system (DNS–[C6&DC]m) based on a C6 cell membrane and dendritic cell (DC) membrane to load DTX for targeting glioma. The prepared nanoparticles had high drug loading ability to significantly increase the drug concentration, and due to the homologous targeting ability of the C6 cell membrane, these nanoparticles could more effectively inhibit the rapid growth of glioma by precise delivery of DTX to the brain tumor site. Additionally, Shao et al.^359^ also developed a bioinspired nanosystem (MSN@RNase A@CM) based on mesoporous silica coated cancer cell membrane (CM) for the delivery of RNase A targeted cancer therapy to show outstanding anticancer effects.

Moreover, due to the capability of precisely regulating the expression of abnormal genes, gene editing technology has a wide range of applications in various cancer. However, there are still challenges in efficient delivery the CRISPR–Cas9 systems. Except from the commonly used physical methods (electroporation and microfluidic membrane deformation methods) and viral vectors, multifunctional nanoparticles with ideal biocompatibility have shown promising applications in delivery CRISPR–Cas9 systems. These multifunctional nanoparticles commonly employ targeted modification strategies to increase the capability of affinity and precisely targeting to cancer cells. For example, Ren et al.^360^ used mucin‐1 (MUC1) aptamer to modify alginate and added with T22‐NLS peptide for multifunctional leukemia targeting CRISPR–Cas9 plasmid delivery system (P@PPM). In this work, MUC1 aptamer could specifically target MUC1 protein overexpressed on cancer cell membranes and the sequence of T22‐NLS peptide could bind affinity with CXC chemokine receptor (CXCR4). These functional modifications endowed P@PPM a dual‐targeting capability for leukemia cells. The prepared P@PPM could enhance CRISPR–Cas9 plasmid delivery into leukemia cell nuclei and obviously reduced the expression of CXCR4 mRNA after gene editing. In addition, the aptamer could also integrate with cell penetrating peptides, such as TAT peptide. Tang et al. developed multifunctional nanoparticles (GTL_A_RC) based on Au NRs modified with TAT peptide and cell‐type‐specific aptamer (Linker‐Apt) for targeted gene editing and cancer therapy.^361^ In this work, due to the specific recognition function of TAT peptide and Linker‐Apt, GTL_A_RC could target delivery sgRNA/Cas9 complex. The Au NRs in GTL_A_RC showed strong photothermal conversion ability for PTT. These results showed that GTL_A_RC could induce efficient cellular uptake by MCF‐7 cells and inhibited the proliferation of tumor cells through combining targeting gene editing and PTT. Similarity, He et al.^362^ applied AS1411 aptamer and TAT peptide to multifunctional‐modified carboxymethyl CS to delivery CRISPR–Cas9 plasmid for downregulation the β‐catenin expression of tumor cells by targeting gene editing.

#### 3.1.2 Environment‐responsive delivery

The tumor microenvironment (TME) refers to the internal environment of tumor cells, including interstitial cells, microvessels, extracellular matrix, and many secreted factors.^363^, ^364^ Compared with normal tissues, the TME has abnormal physiological and biochemical characteristics, such as acidic extracellular pH, hypoxia, and excess GSH. To realize the target TME, multifunctional nanoparticles are designed with sensitive functional groups or bonds to enhance drug release for cancer therapy, such as pH sensitive and redox sensitive.^365^, ^366^, ^367^

*pH sensitive*: The pH‐sensitive drug delivery of multifunctional nanoparticles is widely used in cancer therapy.^368^, ^369^, ^370^ Due to the hypoxia, lactic acid accumulation, and poor perfusion of the TME, the pH value of solid tumors is acidic. The extracellular pH of normal tissue is maintained at 7.4, while the pH of the TME is maintained at 6.5–6.8.^371^ To improve the acidic TME, multifunctional nanoparticles with pH‐responsive bonds can be made to be cleavable in the TME and release drugs with tumor targeting. Multifunctional nanoparticles with the imine bond (C═N─) are prone to hydrolysis under acidic conditions, such as in the presence of amide bonds. For example, Jing et al.^34^ established a pH‐responsive multifunctional nanoplatform (PECL/DA‐Tat‐M) composed of poly(ethylene glycol)–poly(ε‐caprolactone) with a DA‐Tat decoration to deliver 10‐hydroxycamptothecin for improved anticancer efficacy. In the TME, the coupling of amide bond hydrolysis exposes positively charged Tat peptides. PECL/DA‐Tat‐M nanoparticles could promote cell uptake and show high antitumor efficacy, with inhibitory effects on lung metastasis of 4T1 breast tumor. Additionally, the hydrazone bond is similar to the amide bond with a large electron cloud density of the amino group, which is easier to cleave in the TME. Liu et al.^109^ constructed a type of multifunctional nanocarrier (Bio–oHA–Hyd–FA, nanoactiniaes) based on FA and biotin‐conjugated HA (Bio‐oHA) to codeliver icariin (ICA) and curcumin (Cur) for multitargeted therapy for breast cancer. This nanosystem not only enabled targeted drug delivery, but also allowed ICA and Cur to accumulate in tumor tissues through the formation of hydrazine bonds between Bio–OHA and FA. Their results showed that these nanoparticles could enhance the drug release at the TME and effectively suppress tumor volume with pH‐sensitive properties. Due to the amphipathic nature of the imidazole compound, multifunctional nanoparticles modified by imidazole functional groups could be broken in the acidic TME. Indeed, Xu et al.^372^ designed a pH‐responsive multifunctional nanosystem (DOX/IHC nanoparticles) based on imidazole and cholesterol conjugated hydroxyethyl starch (HES) for the delivery of DOX for cancer treatment. Due to the presence of amphiphilic imidazole on the surface, the IHC nanoparticles can effectively load DOX, showing pH‐sensitive release behavior and the sustained drug release of nanoparticles. Their results also showed that the cumulative release of DOX/IHC nanoparticles increased significantly after 100 h with decreasing pH, reaching approximately 50.2 and 67.8% at pH 6.5 and 5.0, respectively.

In addition, mRNA‐based therapy has the advantages of not integrating into the genome and expressing the target protein in the cytoplasm, and has important application prospects in cancer therapy. Due to the instability of mRNA, the difficulty for mRNA applications lies in the development of delivery vectors. However, multifunctional nanoparticles provide a new idea for the application of mRNA. Based on the efficient delivery capability of mRNA, these multifunctional nanoplatforms add pH‐sensitive bonds for enhancing mRNA release at tumor site, such as zinc─ligand bonds. For example, Wang et al.^373^ prepared a pH‐sensitive mRNA delivery platform (DMONs–ZIF‐8) based on zeolitic imidazolate framework‐8 (ZIF‐8) and dendritic mesoporous organosilica nanoparticles. In their design, the large pores of ZIF‐8 were used for enhancing loading capacity of mRNA. The zinc─ligand bonds in DMONs‐ZIF‐8 would be cleaved by acidic pH and promoted endosomal escape. Their results suggested that the prepared nanoparticles enabled successful endosomal escape and higher mRNA transfection efficiency. Similarity, Li et al.^374^ used pH‐sensitive cyclic benzylidene acetal groups to functional modify lipidoid nanoparticles for enhancing mRNA delivery. Moreover, multifunctional nanoparticles could achieve the purpose of pH‐sensitive by chemical modification to adjust the acid dissociation constant (p*K*a value), such as increasing the p*K*a value to release the mRNA in tumor acidic environment. Xiong et al.^375^ designed a multifunctional dendrimer‐based lipid nanoparticle platform (DLNPs) with pH‐sensitive for mRNA delivery and NIR imaging‐guided tumor therapy. In their design, they used tetramethyl‐BODIPY (4,4′‐difluoro‐4‐bora‐3a,4a‐diaza‐s‐indacene) as core and added anilines to tune the p*K*a. With the pH decreased during endosomal maturation, the protonation of aniline bonds in PBD lipid could not only expose the BODIPY core, but also ensure the effective release of mRNA in the tumor microenvironment. Their results demonstrated that DLNPs could efficiently release mRNA and produce 5 to 35‐fold more functional proteins.

*Redox sensitive*: Redox‐sensitive drug delivery systems rely on redox‐sensitive chemical bonds that can be broken by GSH or ROS reduction for precise delivery and rapid drug release.^371^, ^376^, ^377^ Due to the abnormally expressed redox substances in the tumor environment, overexpressed GSH and ROS can act as stimulators and be used in the design of multifunctional nanoparticles with redox‐sensitive bond to enhance drug release. Disulfide bonds are the most commonly used chemical bonds in multifunctional nanoparticle strategies, and following delivery to the TME, they are reduced to sulfhydryl groups at high concentrations of GSH.^140^, ^378^, ^379^ For example, Mousazadeh et al.^80^ developed a host‐guest supramolecular nanoparticle (HGS nanoparticles) based on folate‐appended‐PEI‐β‐CD to deliver adamantane‐conjugated DOX (Ad‐DOX) and human telomerase reverse transcriptase‐small interfering RNA (hTERT siRNA) for targeted cancer therapy. Due to the redox‐sensitive disulfide bond in the system, the drugs could be released into cancer cells in response to their biological environment, showing the comprehensive antitumor properties of DOX‐enhanced gene transfection. Additionally, selenide is a congener of sulfur and the diselenide bond could be oxidized by endogenous H_2_O_2_ to seleninic acid.^380^, ^381^, ^382^, ^383^ Shao et al.^359^ used the cancer cell membrane‐modified selenium‐based MSNs to deliver RNase A (MSN@RNase A@CM) for cancer treatment. These diselenide‐bridged MSNs can encapsulate RNase A into internal pores via electrostatic interactions and release RNase A upon exposure to oxidation or redox conditions in the TME. The results showed that selenium‐based MSNs degraded rapidly after incubation with H_2_O_2_ and GSH conditions and exhibited excellent cancer cell inhibition efficiency. Moreover, thioketals (TK) bonds can be cleaved by ROS in the tumor site, forming a thiol‐containing group and acetone.^384^, ^385^, ^386^ Liao et al.^387^ developed a nanomedicine (M‐TDOX/Lap@TGC) composed of DOX conjugated MSN, β‐Lapachone (Lap), and triphenylphosphonium‐modified CS for improving synergistic oxidation‐chemotherapy. DOX was conjugated with MSNs and formed TK bonds. The loaded Lap‐induced increases in ROS through the quinone oxidoreductase 1 catalytic pathway. Their results showed that compared with the free DOX group and free Lap group, the M‐TDOX/Lap@TGC group showed the most effective inhibitory effect, with a tumor inhibition rate of 78.49% in C666‐1 tumor‐bearing mice. Moreover, others used thioether bonds and Au─S bonds to modify nanoparticles to enhance drug release with a redox response.^280^, ^388^, ^389^, ^390^

### 3.2 Multifunction in therapy

#### 3.2.1 Immunotherapy

Tumor immunotherapy is a treatment method that produces tumor‐specific immune responses through active or passive methods to inhibit and kill tumors.^391^, ^392^ Tumor immunotherapy regulates the polarization of immune cells and the active immune signaling pathway for treatment of cancer with fewer side effects compared with conventional therapy. Based on the drug delivery ability of nanoparticles, the immune activators are codelivered by multifunctional nanoparticles to enhance cancer therapy through promoting an immune response. The small molecular Toll‐like receptor (TLR) agonist is a natural immune system stimulator, which can activate DCs and effector T cells to release proinflammatory factors.^393^, ^394^, ^395^ For example, Wagner et al.^282^ designed a multifunctional nanoplatform (R848‐loaded MSNs) based on MSNs to deliver the TLR7/8 agonist resiquimod (R848) for efficient cancer immunotherapy. In their design, R848 stimulated DC maturation, resulting in the expression of high levels of MHC and costimulatory molecules CD80 and CD86 on the surface of DCs, which promoted the secretion of proinflammatory cytokines leading to an immune response. The PD‐L1 inhibitory peptide could block the combination of PD‐1 on T cells and PD‐L1 on tumor cells, to relieve the inhibition of T cells and resume normal defense function.^396^ Meng et al.^324^ constructed a multifunctional nanoplatform (SPIO NP@M‐P) based on H460 lung cancer cell membranes coated with SPIONs for the targeted delivery of PD‐L1 inhibitory peptide (TPP1) as a cancer immunotherapy. The results showed that the affinity between TPP1 and PD‐L1 was higher than that between PD‐1 and PD‐L1, suggesting that TPP1 is an effective inhibitor of the PD‐1/PD‐L1 interaction to reactivate T cell function. The ability of TPP1 to interfere with PD‐1/PD‐L1 was then applied as a potential drug for tumor immunotherapy. Moreover, ovalbumin (OVA) is a protein antigen that can be taken up by DCs to present exogenous antigens to MHC I via lysosomal escape and further induce a cytotoxic T lymphocyte (CTL) response. Huang et al.^397^ used PDA coated MSNs to deliver model antigen OVA and ammonium bicarbonate ((MSN–ABC@PDA–OVA) for photothermal‐immunotherapy against melanoma. OVA can be effectively delivered to the target site due to the load protection in these nanoparticles, which induces significant immune responses. The prepared nanoparticles significantly reduced tumor‐associated immunosuppression and effectively eradicated primary tumors with a cure rate of 75% for B16‐OVA melanoma in model mice. Additionally, others used multifunctional nanoparticles coated with a DC cell membrane to enhance tumor immune therapy^337^.

#### 3.2.2 Photodynamic therapy

Photodynamic therapy (PDT) is a means of delivering photosensitizers (PS) by vectors to directly irradiate the site with a specific wavelength of laser to produce photochemical reactions.^398^ PDT needs to meet the conditions of visible light, oxygen, and the presence of PS.^399^ Several PS have been approved by the US FDA for cancer therapy, including Porfimer sodium (Photofrin) for bladder cancer treatment and 5‐ALA for actinic keratosis treatment.^400^ When the PS receives light energy, it can transfer the energy to the surrounding oxygen and generate ROS or singlet oxygen (^1^O_2_)^401^. Due to the enhanced permeability and retention effect (EPR effect) of the multifunctional nanoparticles, PS can be delivered to the tumor site and enhance cancer therapy with light energy.^402^, ^403^, ^404^ IR780 is a small molecule of cyanine dye, containing two nitrogen‐contained heterocycles connected by sparse bond.^110^ The nitrogen‐containing heterocycles have charged chromophores that can generate electronic transitions and induce PDT under near‐infrared light irradiation. For example, Zhang et al.^325^ developed a multifunctional nanoparticle (HA&RBCm‐LC nanoparticles) composed of an HA‐hybridized RBC membrane and lipid to deliver of PTX and IR780 for nonsmall cell lung cancer PDT. IR780 could quickly and efficiently produce toxic^1^O_2_ after being irradiated by NIR at 808 nm, and generated ROS for PDT‐induced endogenous mitochondrial apoptosis. Their results showed that the apoptosis rate of A549 cells in the PTX/IR780‐HA&RBCm‐LCNP group treated by NIR was higher than that in the non‐NIR‐treated group, suggesting that IR870‐induced PDT significantly increased tumor apoptosis. Chlorin e6 (Ce6) is obtained from the degradation of chlorophyll a, and has strong absorption in the near‐infrared for deep tissue treatment.^405^, ^406^, ^407^, ^408^ Dapkute et al. prepared photosensitizer Ce6‐modified CdSe/ZnS photoluminescent quantum dots (QDs), which combined cancer diagnostic and PDT abilities.^409^ The energy from excited QDs could be efficiently transferred to Ce6 attached to its surface via the Förster resonance energy transfer mechanism under blue light irradiation, leading to the generation of ^1^O_2_ and improved PDT. Their results illustrated that QD‐Ce6 complexes generated 10‐fold and 19‐fold higher singlet oxygen than Ce6 and QDs in PBS, respectively. Additionally, Ppa, a compound formed by the natural product chlorophyl a with an aromatic azalenene structure, has strong absorption spectra at 600—700 nm.^410^ Zheng et al.^65^ developed a self‐stabilized supramolecular assembly (SSA) based on a PEG‐dendritic peptide‐Ppa (PDPP). Ppa possesses an aromatic azalenene structure, which could generate singlet oxygen and trigger cell death under 660 nm laser irradiation. Their results demonstrated that Ppa exhibited strong cytotoxicity to A549 cells under laser irradiation and was used for PDT. After laser irradiation, the tumor retention and PDT effect of Ppa‐based SSA were significantly enhanced in two highly metastatic 4T1 breast cancer and A549 lung cancer animal models. Moreover, multifunctional nanoparticles with photo sensitivity could be used as photosensitizers to enhance cancer therapy with light energy, such as Pt,^233^ Mo_2_C,^295^ ICG,^411^ red/BP,^318^ and CQDs.^309^

#### 3.2.3 Photothermal therapy

PTT refers to the use of photothermal agents (PTA) to absorb the energy of near‐infrared light and convert it into thermal energy, causing thermal damage and ablation to tumor tissue or cells.^412^ In this process, PTA exhibits a thermal effect and the increased temperature could damage the tumor vasculature and lead to a lack of oxygen and nutrients, causing the change in tumor microenvironment and the death of tumor cells.^334^ The thermal energy is introduced in multifunctional nanoparticles for increasing drug release and cancer therapy with PTT.^413^ To realize PTT, multifunctional nanoparticles with photothermal conversion ability or codelivery with photosensitive molecules can be employed in this delivery system. Moreover, PDA is an organic polymer that can absorb NIR light and improve photothermal conversion ability, while increasing the drug release as ananoparticle vector.^207^, ^414^ Zhang et al.^42^ prepared FA‐modified PDA nanoparticles for ROC1 siRNA delivery with PTT. The prepared nanoparticles not only had the ability to target tumor cells and pH response to effectively deliver ROC1 siRNA, but could also induce the killing of a vast number of cancer cells with the photothermal properties of PDA. Compared with control groups, their results showed that PDA nanoparticles improved drug release to 26% by thermal expansion efficacy. After knocking down the ROC1 gene, PDA nanoparticles could inhibit the growth of Huh7 cells increasing to 55%. Additionally, Au nanoparticles have the ability to localize surface plasmon resonance (LSPR) absorption, which can convert light energy into heat energy by nonradiative relaxation.^415^, ^416^, ^417^ Pulagam et al. designed multifunctional nanoparticles ([^64^Cu]AuNR–mPEG@[4]^−^) based on ^64^Cu‐labeled Au NRs modified by boron‐based anion and PEG (mPEG) for PTT for cancer.^169^ Due to LSPR absorption and scattering of AuNRs, light energy is converted into heat energy by nonradiative relaxation under NIR light, resulting in cell death. Their results showed that under the irradiation of 808 nm NIR laser for 48 h, the local temperature of the 3D cell model incubated with AuNRs significantly increased to 23°C and the cell viability was obviously decreased. Additionally, ICG is a near‐infrared dye with an ethenyl indole structure that has been approved by the US FDA for clinical diagnostic imaging with good photothermal conversion efficiency.^418^ Zhou et al.^419^ designed a HA‐modified multifunctional microneedle (ZIF–ICG@ZIF–GOx–Cat@HA MNs) based on a ZIF‐8 to codeliver GOx, catalase (Cat), and ICG for cascade reaction‐enhanced cancer therapy. The stability of GOx and Cat was improved due to the spatial limitations of ZIF‐8. The results showed that tumor cells were more sensitive to ICG‐guided PTT through the down‐regulation of heat shock protein induced by Gox, while the local tumor temperature was rapidly increased to inhibit the growth of tumor cells following intravenous injection.

#### 3.2.4 Sonodynamic therapy

SDT is a type of PDT that uses low‐frequency low‐intensity ultrasound to irradiate a sonosensitizer to generate ROS to kill tumor cells.^420^ The treatment mechanism of SDT mainly includes generating ROS, acoustic cavitation, and inducing tumor apoptosis.^421^ SDT is a noninvasive treatment method that can deeply penetrate the tumor with fewer side effects, showing excellent development potential in the field of tumor treatment.^422^, ^423^, ^424^ Moreover, ultrasound has also been introduced in multifunctional nanoparticles to increase tumor apoptosis and enhance cancer therapy with SDT. Multifunctional nanoparticles could be codelivered with a sonosensitizer to realize SDT.^425^, ^426^, ^427^ Hematoporphyrin monomethyl ether (HMME) is a porphyrin derivative with a tetrapyrrole ring structure that could generate highly toxic singlet oxygen under ultrasound irradiation. Huang et al.^428^ prepared PLGA‐encapsulated melanin nanoparticle and HMME nanoparticles with FA modification for PAI‐guided SDT. The tetrapyrrole ring structure of HMME enables it to absorb energy and produce highly toxic singlet oxygen under ultrasound irradiation. After treatment with FHMP‐nanoparticles for 15 days, the nanoparticles exhibited high tumor growth rate in MDA–MB‐231 tumor‐bearing mice and combined US irradiation with PAI for cancer therapy. Additionally, multifunctional nanoparticles could be codelivered with TiO_2_, in which the defect in the TiO_2_ lattice acts as an electron trap site to oxidize H_2_O to generate •OH and ^1^O_2_ under US irritation.^202^ Tao et al.^429^ reported a multifunctional cascade nanozyme (HABT‐C) with TiO_2_ nanosphere, which was modified by Au nanoparticles and a carbon dot to enhance the effect of SDT, alleviate hypoxia, and reverse immunosuppression for cancer therapy. The defect in the TiO_2_ lattice acted as an electron trap site to oxidize H_2_O to generate •OH and ^1^O_2_ under US irradiation, resulting in oxidative damage to cancer cells. Their results showed that the tumor growth of the HABT‐C group with US irradiation was effectively suppressed after 16 days of treatment, and no obvious tumor recurrence was observed in the tumor xenograft mouse model. Moreover, multifunctional nanoparticles could be codelivered with an MOF based on a porphyrin derivative as an organic ligand, which could convert the surrounding oxygen into ^1^O_2_ under US stimulation.^430^ Zhang et al.^431^ prepared a dual‐sonosensitizer nanoparticle (Zr–MOF@AIPH nanoparticles) based on a zirconium MOF loaded with 2,2‐azobis[2‐(2‐imidazolin‐2‐yl) propane] dihydrochloride (AIPH) for photoacoustic, fluorescence, and ultrasound imaging‐guided SDT. As a porphyrin‐based sonosensitizer, Zr–MOF could convert the surrounding oxygen to ^1^O_2_ under US stimulation. AIPH could generate alkyl radicals under hypoxia. The generation of singlet oxygen and oxygen‐irrelevant radicals enabled strengthened SDT efficiency. Their results showed that the Zr–MOF@AIPH with US had an obvious inhibitory effect on tumor growth with a tumor inhibition rate of 41.9%.

#### 3.2.5 Chemodynamic therapy

CDT is a new type of minimally invasive therapy for tumor treatment that is based on the acidic tumor environment, in which the overexpressed H_2_O_2_ is catalyzed by transition metals, causing a Fenton or Fenton‐like reaction in tumor cells.^432^, ^433^ The Fenton reaction refers to a classical reaction in which H_2_O_2_ is catalyzed by ferrous ions (Fe^2+^) to generate hydroxyl radical (•OH). The Fenton‐like reaction refers to a reaction process in ferric iron (Fe^3+^) or other transition metals (such as Cu, Mn, and Ag), which accelerate or replace Fe^2+^ to catalyze H_2_O_2_.^434^, ^435^ Multifunctional nanoparticles are introduced with CDT, which could enhance cancer therapy with Fenton or Fenton‐like reactions.^436^ In detail, multifunctional nanoparticles could be codelivered with metal materials for achieving CDT, such as MnO_2_,^437^ CuAS,^438^ Fe_3_O_4_, and Zn–Mn–Ferrite^439^. For example, Zhao et al.^440^ designed a multifunctional yolk–shell nanosystem (Fe_3_O_4_@C/MnO_2_–PGEA, FCMP) composed of iron oxide (Fe_3_O_4_) cores, C/MnO_2_shells and polycationic cloak (CD–PGEA) to deliver the anticancer gene p53 for MRI‐guided multiaugmented CDT and synergistic tumor treatment. In their design, the reduction of MnO_2_ was triggered by GSH to produce Mn^2+^, and the FCMP nanohybrid partially dissociated the Fe_3_O_4_ nucleus to release iron ions, which reacted with endogenous hydrogen peroxide (H_2_O_2_) to produce toxic hydroxyl radicals (·OH). Their results showed that under an external magnetic field, the most significant tumor suppressive effect was observed following intravenous injection of the FCMP/p53+NIR group after 10 days. Similarly, Lin et al.^251^ designed a multifunctional nanoplatform (MS@MnO_2_ nanoparticles) based on MN dioxide (MNO_2_)‐coated mesoporous silica to deliver CPT for MRI‐monitored chemo‐chemodynamic combination cancer therapy. In their results, the tumor growth in U87MG tumor‐bearing mice was significantly inhibited and showed higher rates of apoptosis than control groups after intravenous injection of MS@MnO_2_ nanoparticles. Moreover, Lee et al.^441^ reported using copper (II) arsenite (CuAS) coupled in the core domains of poly (ethylene glycol)‐*b*‐poly(3,4‐dihydroxy‐*L*‐phenylalanine) to form CuAS‐PMs for CDT treatment of cancer.

#### 3.2.6 Synergistic therapy

Tumor cells are complex, diverse, and specific, leading to enormous challenges in treatment. At present, clinical anticancer research focuses on transforming monotherapy to a synergistic therapy of multiple treatment modality.^442^ The multifunctional nanoparticle delivery system integrates the treatment methods of monotherapy into a platform to obtain synergistic therapeutic effects, which greatly increases their anticancer ability. The complementary properties of these synergistic therapy strategies can compensate for the insufficiency of monotherapy.^443^

*PDT/PTT*: PDT requires high volumes of oxygen. However, the excessive consumption of oxygen will make the tumor excessively hypoxic, weakening the therapeutic effect of PDT. To improve the efficacy of PDT, multifunctional nanoparticles could be used to codeliver PTT photosensitizer to improve the ROS production capacity of the photosensitizer and increase the oxygen permeability, such as metal materials with photosensitizer and material with nonmetal material.^444^, ^445^, ^446^, ^447^, ^448^ Based on drug delivery ability, Au is an excellent PTT material that can be codelivered with PS, such as Ce6. For example, Kim et al. prepared a multifunctional nanoplatform (Ce6–AuNP–Lf) based on lactoferrin‐coated Au nanoparticles to deliver Chlorin e6 (Ce6) for the treatment of glioblastoma multiforme with phototherapy.^177^ The PDT photosensitizer Ce6 was proximately conjugated on the surface of the PTT agent AuNP, leading to plasma crossover between Ce6 and AuNP that greatly improved the ROS production capacity of Ce6. Their results illustrated that Ce6–AuNP–Lf could effectively intervene in the progression of glioma and suppress the growth of tumors in a subcutaneous GBM mouse model by first applying PDT (671 nm), followed by PTT (532 nm). Additionally, multifunctional nanoparticles with dual roles of PTT and PDT could be used as PDT/PTT, such as black phosphorous nanosheets. Zhang et al.^317^ designed multifunctional nanoparticles (HA–BP) based on BP modified by HA for the combination therapy of cancer. With the modification of HA, HA–BP could target cancer cells and reverse TAMs to the M1 type. Due to the high photothermal conversion efficiency and ROS capacity of BP, the prepared nanoparticles could induce ICD‐mediated antitumor immunity through phototherapy in vitro and in vivo.

*PDT/CDT*: As CDT can catalyze H_2_O_2_ to generate oxygen through Fenton and Fenton‐like reactions, CDT can provide high volumes of oxygen for PDT and enhance the generation of hydroxyl radicals (•OH).^449^, ^450^ Multifunctional nanoparticles can catalyze H_2_O_2_ to enhance PDT with CDT and are multifunctional based on the fact that metal nanoparticles exhibit excellent catalysis for codelivery with photosensitizers, such as ICG and Ce6. For example, Chen et al.^451^ reported the self‐assembly of Ce6 and hemin into multifunctional nanoparticles (HC nanoparticles) for PDT/CDT/Ferroptosis combination therapy. The PDT reagent Ce6 could convert O_2_ produced by hemin catalysis H_2_O_2_ into ^1^O_2_ to enhance PDT efficiency. Hemin induces GSH to release Fe^3+^ and redox to Fe^2+^ for effective CDT through the Fenton reaction. These results suggest that a low dose of HCNP nanoparticles may effectively destroy cancer cells and inhibit tumor growth under 660 nm laser irradiation.

*PTT/CDT*: Due to the limited transmission ability of laser light, PTT cannot penetrate into the deep layer of the organism and is still difficult to implement in monotherapy with PTT. Based on the photothermal conversion ability of PTT, multifunctional nanoparticles are introduced chemodynamics to enhance the photothermal performance by catalyzing H_2_O_2_ molecules.^452^, ^453^, ^454^ For example, Li et al.^188^ designed a multifunctional nanosystem (BCG) based on Cu‐modified black phosphorous quantum dots (BPQDs) to load glucose oxidase (GOD). BPQDs released Cu^2+^ to induce GSH depletion, and transformed Cu^2+^ into cuprous ions for CDT. GOD consumes glucose to produce H_2_O_2_ and enhance CDT, and BPQDs capture Cu^2+^ to enhance the photothermal performance. In vitro and in vivo experiments showed that this combined PDT and CDT strategy had remarkable anticancer effects.

**FIGURE 5:**
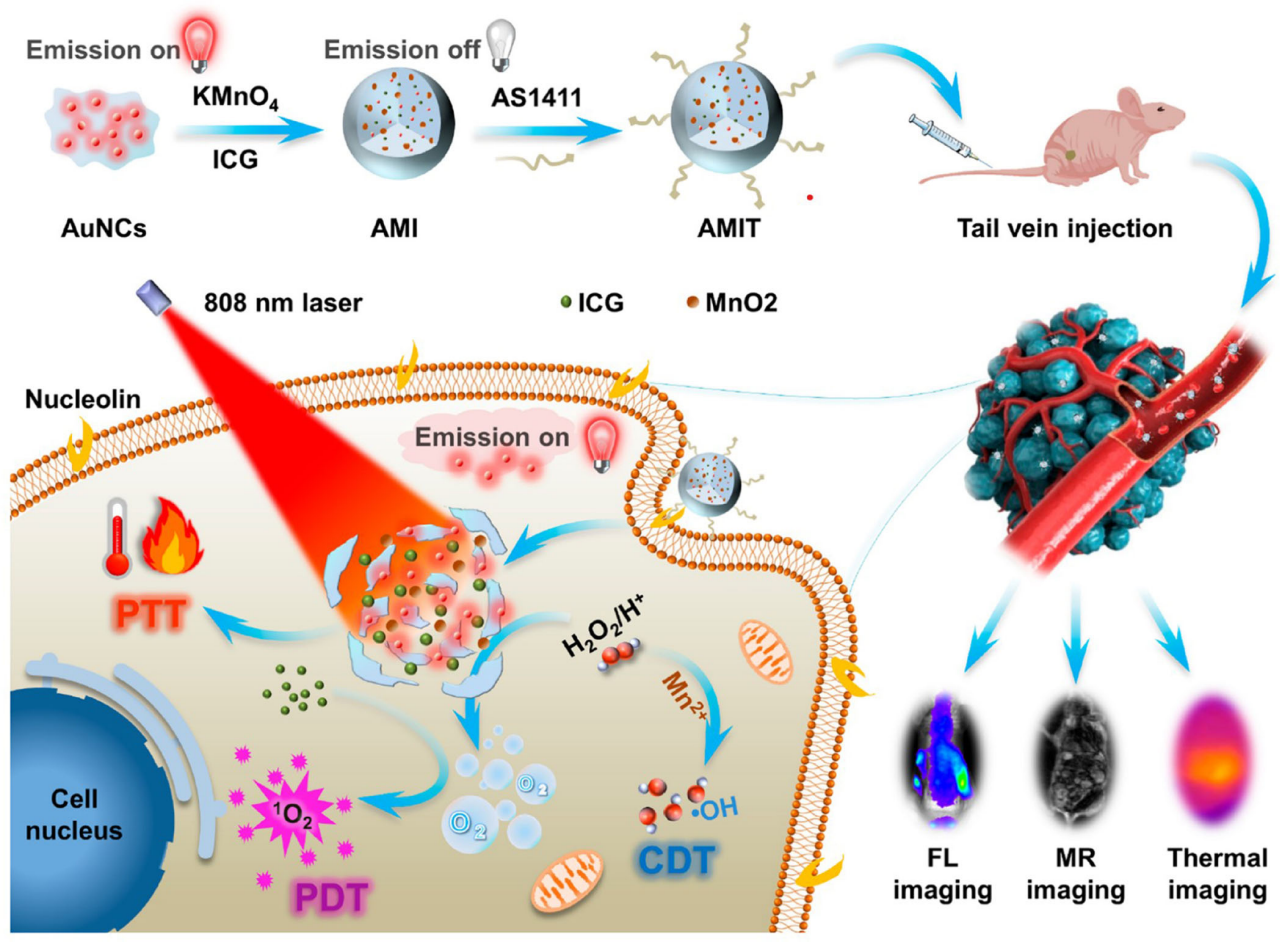
Scheme illustrating of constructed multifunctional nanoplatform (AMIT) based on gold nanoclusters (AuNCs) coated with manganese dioxide (MnO_2_) to codeliver ICG and the aptamer AS141 for targeting and image‐guided PDT/CDT/PTT. Reprinted with permission from Ref. [458], Copyright 2021 American Chemical Society.

**FIGURE 6:**
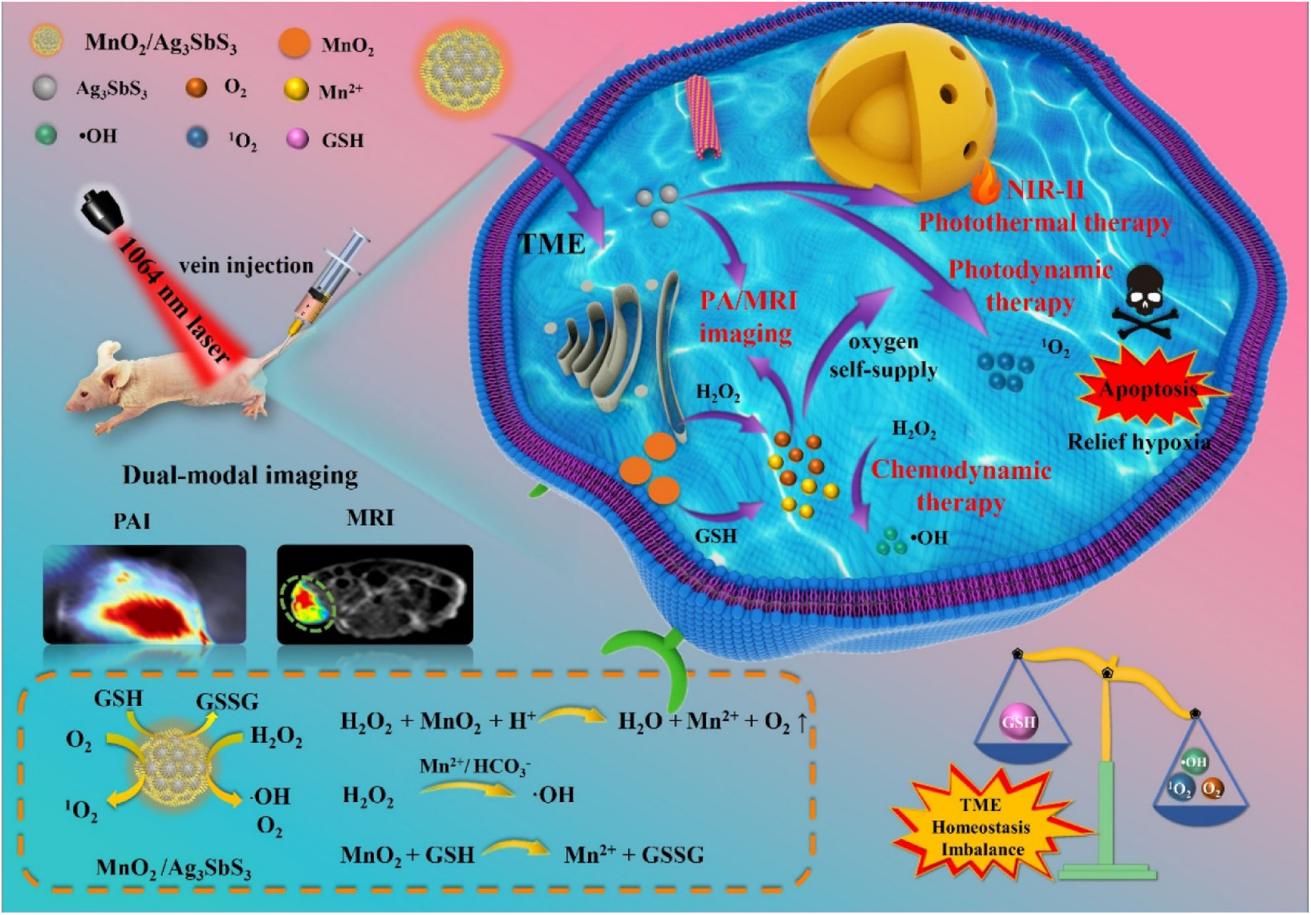
Scheme illustrating of a multifunctional nanoplatform (MA) based on manganese dioxide‐coated Ag_3_SbS_3_ (PTT and PDT agent) for cancer treatment with PDT/CDT/PTT under a single NIR‐II laser. Reprinted with permission from Ref. [456], Copyright 2022 American Chemical Society.

*CDT/PTT/PDT*: CDT/PTT/PDT can also be combined for trimodal synergistic therapy. Due to the tissue penetration ability of PTT, CDT can promote the production of oxygen and can generate singlet oxygen with strong activity with PDT, achieving effective tumor killing effect in deep tissues. The multifunctional nanoparticles are introduced with light/heat/physical dynamics for cancer therapy with synergistic therapy.^455^, ^456^, ^457^ For example, Sun et al.^458^ constructed a multifunctional nanoplatform (AMIT) based on Au nanoclusters (AuNCs) coated with MnO_2_ to codeliver ICG and the aptamer AS141 for targeting and image‐guided PDT/CDT/PTT (Figure 5). The TME‐responsive dissociation of MnO_2_ could produce O_2_ to enhance ICG‐guided PDT and produce Mn^2+^ for Fenton‐like reaction‐mediated CDT. The treatment efficacy of PDT/PTT could be improved by codelivery with ICG and AuNCs, causing deep tumor penetration. Their results showed that the weight and size of the AMIT group was much smaller than those of the other groups after 21 days. Additionally, Wang et al.^456^ designed a multifunctional nanoplatform (MA) based on MNO_2_‐coated Ag_3_SbS_3_ (PTT and PDT agent) for cancer treatment with PDT/CDT/PTT under a single NIR‐II laser (Figure 6). Their results showed that the tumor growth was inhibited or even eliminated in the MA group under 1064 nm laser.

### 3.3 Multifunction in imaging

#### 3.3.1 Magnetic resonance imaging

MRI uses hydrogen protons in tissue to form magnetic moments and excitation by a pulsed radiofrequency evacuation.^459^ Subsequently, the hydrogen proton will release the excitation energy received by radiofrequency coils and convert it to a grayscale image.^460^ MRI does not require injection of contrast agents, has no ionizing radiation, and has no adverse effects on the body, with excellent potential advantages for disease diagnosis.^461^, ^462^, ^463^ The multifunctional nanoparticles are introduced ferromagnetism or paramagnetic for enhancing tumor therapy with MRI‐guiding.^464^, ^465^, ^466^ There are some MRI contrast agents that have been used in clinical or are undergoing clinical trials based on multifunctional backbone for treatment cancer. For example, SPIONS is injected to track the drugs for MRI cellular imaging of hepatic parenchyma (NCT04682847); Ferrotran^®^ (a Fe_3_O_4_ preparation) is used to virtual histology of the bladder wall for bladder cancer staging (NCT04369560); and ultrasmall superparamagnetic iron oxide (USPIO) nanoparticles are applied for measuring the activity of innate immune system within MS lesions (NCT05357833). Meanwhile, multifunctional nanoparticles containing SPIONs showed a typical darkening property under static magnetic fields because of the short transverse relaxation time (T_2_) of protons.^165^, ^467^ Thirunavukkarasu et al.^26^ prepared MIDEN with SPIONs (Fe_3_O_4_, SPIONs) and DOX for MRI‐guided thermo‐chemotherapy. In their design, SPIONs showed a typical darkening property under static magnetic fields due to the short transverse relaxation time (T_2_) of protons and demonstrated that MIDEN could be used in MRI. After intratumoral injection of MIDEN in CT26 tumor‐bearing mice, MRI signals were homogenously observed in the tumor region to achieve tumor imaging. Due to the reduced antiferromagnetic interaction, SPION multifunctional nanoparticles can be substituted by nonmagnetic atoms to enhance the MRI signal. For example, Gondan et al. prepared mZnSPION–polyIC–R837 based on PEGylated phospholipid‐encapsulated Zn*_x_* Fe_3‐_ *_x_* O_4_ nanoparticles to deliver polyIC and the TLR7 agonist imiquimod (R837) for MRI and antitumor immunity effects.^468^ In their design, the overall magnetization and the spin–spin relaxation rate (R_2_) of SPION could be optimized and significantly increased by zinc (II) doping, resulting in the improved MRI detection sensitivity of SPION. They illustrated that mZnSPION exhibited a strong MRI contrast effect with a transverse relaxivity (*r*_2_) that was three times larger than mSPION. Due to the paramagnetic relaxed ability, multifunctional nanoparticles containing Mn^2+^ could be used for MRI with T_1_ signals.^469^ Moreover, Chen et al.^470^ reported a biomimetic virus‐like nanocomplex (SDN) composed of photosensitizer Ce6‐loaded nanostructured lipid carrier and poly (allylamine hydrochloride)‐functionalized MnO_2_ nanoparticles. In their design, Mn^2+^ could be released quickly due to the presence of intracellular redox components. The SDN nanocomplex exhibited strong T_1_ MRI signals, which were enhanced by Mn^2+^.

#### 3.3.2 Fluorescence imaging

FI uses a specific wavelength of laser light to irradiate fluorophores with fluorescent emission properties, absorb the light energy, and emit light for biological imaging.^471^ FI can be performed in vivo without puncturing the skin, and has the advantages of less influence on biological tissue, strong tissue penetration, and a high signal‐to‐noise ratio.^472^ Multifunctional nanoparticles with fluorescent emission properties can be used in FI with real‐time tracking of drug delivery and release.^473^, ^474^, ^475^ In detail, multifunctional nanoparticles can be codelivered with bioactive agents containing luminescent groups to form nano prodrugs, such as dioxetane.^476^ The dioxetane is unstable after being irradiated by NIR and forms two ketone structures located in the ground state and the excited state, respectively.^477^ When the excited ketone structure transitions back to the ground state, fluorescence is generated. For example, He et al.^478^ developed an organic afterglow protheranostic nanoassembly (APtN) for activating both pharmaceutical effects and diagnostic signals in the TME. The phenylboronic ester group of APtN could be cleaved by H_2_O_2_ to uncage the afterglow substrate and react with silicon 2,3‐naphthalocyanine bis(trihexylsilyloxide) (NCBS) to form PEG–dioxetane. Due to the instability of PEG–dioxetane, fluorescence was generated under NIR. Their results showed that the afterglow of APtN in solution could be increased 820‐fold after treatment with H_2_O_2_, causing permitting the real‐time in vivo feedback for the status of prodrug activation. Additionally, multifunctional nanoparticles can be codelivered with persistent luminescence nanoprobes (such as rare‐earth‐doped nanoparticles), which can store photons in the traps by irradiating them with ultraviolet (UV) light and subsequently releasing the stored energy under thermal energy excitation. Kong et al.^479^ reported a persistent luminescence nanoprobe (TRZD) for the NIR imaging and therapy of glioma. Due to the Cr^3+^/Sn^4+^ codoped ZnGa_2_O_4_, TRZD could cause persistent luminescence in NIR imaging under thermal energy excitation. Their results demonstrated that the luminescence of TRZD could last longer than the 7200 s and still maintain comparable intensity, and luminescence could also be renewed by UV light. Additionally, multifunctional nanoparticles with aromatic amino acids are intrinsically fluorescent under near‐infrared light, such as tryptophan, tyrosine, and phenylalanine. Fan et al.^142^ prepared an NIR fluorescent peptide nanoparticle (RGD–f‐P nanoparticles/EPI) with RGD and EPI for cell FI and target drug delivery. In their design, the cyclo [‐(_D_‐Ala‐_L_‐Glu‐_D_‐Ala‐_L_‐Trp)_2_‐] peptide structure of f‐P nanoparticles enabled it to emit visible and NIR fluorescence under 370 and 760 nm laser irradiation. Zinc coordination‐limited energy dissipation during thermal relaxation pathways to obtain better quantum yield and fluorescence intensity. Their results showed that drug delivery to tumor sites could be monitored in vivo by the NIR fluorescence of RGD–f‐P nanoparticles/EPI.

#### 3.3.3 Photoacoustic imaging

PAI uses the photoacoustic effects of biological tissue to perform imaging.^480^ The imaging principle is that when biological tissue is irradiated by a pulsed laser, absorbed light energy, slight local heating and rapid thermal expansion results. This region of the biological tissue expands outward and generates acoustic waves to form a photoacoustic signal.^481^, ^482^ The advantages of PAI include high resolution, strong contrast, and deep penetration, with excellent development potential in cancer therapy.^483^, ^484^ Multifunctional nanoparticles are added with ultrasound for tracking drug release with PAI‐guided cancer therapy.^485^ In detail, multifunctional nanoparticles with light absorption and high photothermal conversion efficiency could achieve PAI, such as inorganic materials (BP quantum dots). Black phosphorous quantum dots with unique layered structures could absorb laser light to convert heat energy and store the energy for PAI. For example, Li et al. prepared a multifunctional Fenton nanocatalyst (BCG) composed of Cu‐doped BPQDs and GOx for cancer therapy with PAI guidance.^188^ Due to its unique layered structure and layer‐dependent bandgap under 808‐nm laser NIR, the BCG in BPQDs possessed strong absorption in the NIR region and allowed for the PAI. Their results suggested that PAI intensity was concentration‐dependent and the PA signal was improved in the BCG NP solution with a concentration of 1 mg/ml. Additionally, multifunctional nanoparticles with photothermal conversion capabilities can also be used for PAI due to their ability to induce thermoelastic and ablative generation of ultrasound, such as PDA. Zhang et al.^486^ proposed the use of acorn‐like Janus nanoparticles (PAA–mCaP/PDA–PEG J nanoparticles) based on mesoporous calcium phosphate for the synergistic treatment of cancer with PAI‐guided chemo‐phototherapy. PDA possessed strong NIR absorption and high photothermal conversion efficiency under 808‐nm laser illumination. Their results showed that the PA signal intensity increased gradually, reaching a peak after intravenous injection of these nanoparticles for 24 h in HepG‐2 tumor‐bearing mice. Moreover, multifunctional nanoparticles codelivered with metal materials can also induce PAI via photo‐induced charge transfer, such as Mo (VI). Wang et al.^487^ reported a multifunctional nanoenzyme (*Ox*‐POM@Cu) based on a polyoxometalate doping with Cu ions for NIR‐II PAI‐guided chemodynamical and PTT. The photo‐induced charge transfer of Mo could realize PAI under 1064 nm laser illumination, leading to an increase in their absorbance in the NIR‐II region. Their results showed that the PA signal was the strongest in tumor tissue after injection of these nanoparticles for 4 h in 4T1 tumors.

#### 3.3.4 X‐ray computerized tomography imaging

X‐ray computerized tomography (CT) imaging is a medical imaging technique that uses the absorption properties of tissue under ray energy to realize tomographic imaging and visualize interior features of tissue.^488^ According to the structure and position of object, the intensity of X‐ray energy could decrease and reflect as the grayscale images. CT imaging exhibits the great advantages of painless, noninvasive, and clear information. In addition, CT imaging is widely applied in cancer treatment and some iodinated contrast medium (ICM) are approved by US FDA, such as iohexol and iodixanol.^489^ Multifunctional nanoparticles modified with large X‐ray attenuation coefficient could monitor drug effect and apply to CT imaging‐guide cancer therapy. Due to the high electron density and atomic number, metallic nanoparticles show large X‐ray attenuation coefficient used for CT contrast agents, such as Au, bismuth, barium, and gallium.^490^, ^491^ For example, Wang et al.^445^ proposed a multifunctional nanosystem (Cu_3_BiS_3_–PEG–(Ce6–Gd^3+^)–FA NPs) using Cu_3_BiS_3_ NPs to anchor FA and chlorin e6 with Gd^3+^ for dual‐modal CT and MR imaging guided cancer treatment. Bismuth element possessed excellent near infrared photothermal conversion performance and large X‐ray attenuation coefficient, which made the prepared nanoparticles have excellent CT imaging performance. Their results indicated that these nanoparticles had a high X‐ray absorption coefficient of 17.7 HU mmol Bi per L and the contrast of CT signal in the tumor was significantly enhanced after 4 h. Similarly, Zhao et al.^270^ also designed a Bi_2_S_3_‐based multifunctional nanoparticle (FBPD NPs) for CT/PA dual‐mode imaging‐guided collaborative therapy of ovarian cancer. Moreover, iodides are small molecular with strong attenuation of X‐ray absorption and have great compatibility in the body, which is usually used as contrast agent, such as iodinated nanoparticles. Fu et al. prepared LC@I‐PANi nanoparticles through the combination of iodic acid and aniline monomers for CT imaging and PA imaging‐guided PTT.^492^ In their design, iodine has a high atomic number, and the introduction of iodinated materials could produce image contrast due to different photoelectric absorption for enabling CT imaging. Their results showed that a strong CT signal was generated at the tumor site after intratumoral injection of LC@I‐PANi in 4T1 tumor‐bearing mice and LC@I‐PANi possessed good biocompatibility in the body.

#### 3.3.5 Multimodal imaging

**FIGURE 7:**
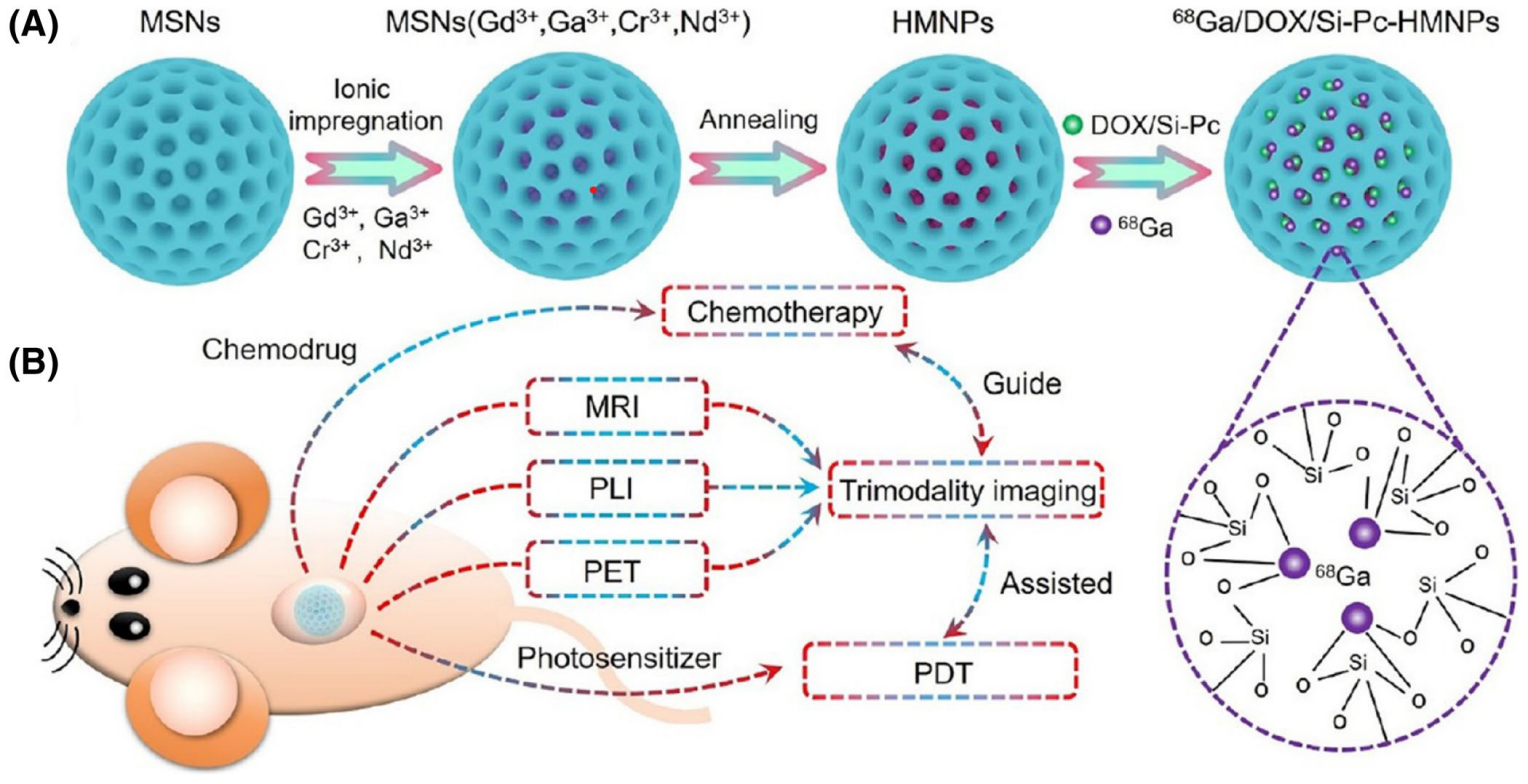
(A) The preparation of of ^68^Ga/DOX/Si‐Pc‐HMNPs based on multifunctional mesoporous nanoparticles. (B) ^68^Ga/DOX/Si‐Pc‐HMNPs could act as an “all‐in‐one” nanotheranostic tool for multimodal imaging‐guided PDT and chemotherapy. Reprinted with permission from Ref. [500], Copyright 2021 American Chemical Society.

Multimodal imaging is the organic superposition of multiple modal image information, which is better used in clinical research and treatment. Multimodal imaging has high sensitivity and resolution, which solves the limitations of a single imaging method and expands tumor imaging methods.^493^, ^494^, ^495^ In this method, multifunctional nanoparticles are added with multiple imaging capabilities to track the process of cancer therapy by multimodal imaging.^496^ In detail, multifunctional nanoparticles with ferromagnetism or paramagnetic for MRI can be combined with photosensitizers for FI and PAI, such as Fe_3_O_4_ codelivery with MnO_2_ and Ce6. The Fe^3+^ and Mn^2+^ ions have the short transverse relaxation time (T_2_) of protons for MRI, and Ce6, with a tetrapyrrole ring structure, can absorb the NIR light for FI. Fan et al.^497^ developed a multifunctional nanoplatform (Fe_3_O_4_@MnO_2_–CSL/Ce6) composed of Fe_3_O_4_ nanoparticles with MnO_2_ coated on the surface, with celastrol and Ce6 for multimodal imaging‐guided chemo‐PDT. Due to the Fe^3+^/Mn^2+^ ions and Ce6, these nanoparticles can significantly improve the magnetic resonance signal and have been used in FI of Bel‐7402 tumor‐bearing mice. The LSPR of Fe^3+^ and Mn^2+^ enabled Fe_3_O_4_@MnO_2_–CSL/Ce6 for PAI under 850 nm wavelengths. Additionally, multifunctional nanoparticles could be codelivered with PFP, which could expand them from nanoscale nanobubbles to microbubbles to achieve the magnetic droplet vaporization (MDV) effect under alternating magnetic field (AMF) irradiation, resulting in further enhancement of USI. Wang et al.^498^ reported magnetic nanodroplets (Fe/Art‐Lip@PFP) based on Fe_3_O_4_ and PFP encapsulated‐liposomes and artesunate (Art) for enhancing the antitumor efficacy of ferroptosis with the guidance of multimodal imaging. The LSPR of Fe_3_O_4_ was useful for PA imaging under 690 nm wavelengths, and the results showed that the PA signal increased linearly with increasing concentrations of MNDs. MNDs filled with PFP could achieve a MDV effect under AMF irradiation to enhance USI. Moreover, multifunctional nanoparticles grafted with radionuclide that have paramagnetic properties and a photothermal conversion ability can be used for MRI/PET/FI. The Gd^3+^ radionuclides emitted positrons during its decay and could be used in PET imaging. Zou et al.^499^ developed multifunctional mesoporous nanoparticles (^68^Ga/DOX/Si‐Pc‐HMNPs) composed of Ga_2_O_3_ (Cr^3+^, Nd^3+^), Gd_2_O_3_, and ^68^Ga for multimodal imaging‐guided PDT and chemotherapy (Figure 7). Their results showed that the longitudinal relaxivity (*r*_1_) of HM nanoparticles was estimated to be 8.908 mM^−1^ s^−1^, higher than that of a commercial positive contrast agent, indicating that HM nanoparticles are effective MRI‐positive contrast agents. After intravenous injection of HM nanoparticles with UV lamp excitation, NIR‐PL signals were detected in the whole LNCaP tumor‐bearing mouse within 5 min. The ^68^Ga of HM nanoparticles emitted positrons during their decay and could therefore be used in PET imaging.

## 4 CONCLUSIONS AND PERSPECTIVES

Recently, researchers have made outstanding progress on the development of multifunctional nanoparticles for cancer therapy. The construction potential of numerous backbones, both organic and inorganic, has been thoroughly evaluated. Moreover, strategies for introducing functional groups or agents to the backbone have also been investigated. As a result, a growing number of anticancer nanoparticles with extensive and synergistic multifunctions have emerged. However, several issues not included in this review remain. As for construction strategies, existing nanomaterials remain insufficient to provide enough docking sites for extension of function. Backbones made from natural products such as proteins, cells, and microorganisms may be exploited in the future. More hybrid backbones are also expected to achieve optimized properties. For example, the organic nanoparticles are expected to be combinated with inorganic nanoparticles. The biodegradability and biocompatibility of organic nanoparticles could provide better delivery capability for inorganic nanoparticles, while inorganic nanoparticles might offer multiple bioimaging functions. Moreover, due to high drug loading (up to 100%) and excellent optical properties, carrier‐free nanoparticles and semiconducting polymer nanoparticles have great potential in constructive multifunctional nanoparticles, and more attention should be paid.

Regarding function design, the application of multifunction nanocarriers should be further expanded rather than just limited to drug delivery or imaging. Promising biomimetic functions, such as recognition, repair, and biomanufacturing, are potential choices for functional enrichment. In addition, to further enhance their clinical potential, it is important to develop more advanced formulations with existing multifunctional nanoparticles. We also suggest paying extra attention to the potential drug production issues for further pharmaceutical development, including formulation, quality control, the availability of raw materials. It is also important to consider in vivo safety studies of multifunctional nanoparticles to obtain more evidence for further clinical application. Despite these limitations, multifunctional nanoparticles exhibit high potential in the context of cancer therapy. More advanced and exciting strategies are expected to be developed in the near future.

## AUTHOR CONTRIBUTION

Y. G., K. Y. W., and J. Z. drafted this manuscript and prepared the figures. X. M. D., Q. S., and K. M. designed the framework for the entire manuscript, provided detailed guidance, and financial support. All authors have checked the manuscript and agree to be publication.

## CONFLICT OF INTEREST

There is no conflict of interest to declare.

## ETHICS STATEMENT

The authors declare that human ethics approval was not needed for this study.

## Data Availability

Not applicable.
